# Prediction of Dichloroethene Concentration in the Groundwater of a Contaminated Site Using XGBoost and LSTM

**DOI:** 10.3390/ijerph19159374

**Published:** 2022-07-30

**Authors:** Feiyang Xia, Dengdeng Jiang, Lingya Kong, Yan Zhou, Jing Wei, Da Ding, Yun Chen, Guoqing Wang, Shaopo Deng

**Affiliations:** State Environmental Protection Key Laboratory of Soil Environmental Management and Pollution Control, Nanjing Institute of Environmental Sciences, Ministry of Ecology and Environment of China, Nanjing 210042, China; feiyangxia28@163.com (F.X.); jiangdengdeng@nies.org (D.J.); kly@nies.org (L.K.); zhouyan@nies.org (Y.Z.); weijing@nies.org (J.W.); dingda@nies.org (D.D.); chenyun@nies.org (Y.C.); nies.sepa@163.com (G.W.)

**Keywords:** contaminated site, groundwater, dichloroethene, natural attenuation, machine learning, LSTM, XGBoost, SHapley Additive exPlanations

## Abstract

Chlorinated aliphatic hydrocarbons (CAHs) are widely used in agriculture and industries and have become one of the most common groundwater contaminations. With the excellent performance of the deep learning method in predicting, LSTM and XGBoost were used to forecast dichloroethene (DCE) concentrations in a pesticide-contaminated site undergoing natural attenuation. The input variables included BTEX, vinyl chloride (VC), and five water quality indicators. In this study, the predictive performances of long short-term memory (LSTM) and extreme gradient boosting (XGBoost) were compared, and the influences of variables on models’ performances were evaluated. The results indicated XGBoost was more likely to capture DCE variation and was robust in high values, while the LSTM model presented better accuracy for all wells. The well with higher DCE concentrations would lower the model’s accuracy, and its influence was more evident in XGBoost than LSTM. The explanation of the SHapley Additive exPlanations (SHAP) value of each variable indicated high consistency with the rules of biodegradation in the real environment. LSTM and XGBoost could predict DCE concentrations through only using water quality variables, and LSTM performed better than XGBoost.

## 1. Introduction

In 2015, the United Nations proposed the Sustainable Development Goals (SDGs) which aim to end poverty, protect the planet, and ensure that by 2030 all people enjoy peace and prosperity. Improving water quality by reducing pollution was one target of SDG goal 6. Protecting water resources has been an eternal goal for humans, while many pollutants coming from agriculture and industries are damaging to water quality. As an important chemical raw material and organic solvent, chlorinated aliphatic hydrocarbons (CAHs) are widely used in agriculture and industries [1]. Due to improper storage and disposal, CAHs could enter the groundwater through volatilization, leakage, and discharge, and have been investigated in many studies [2,3,4,5].

The prevalence of CAHs in groundwater and the hazardous nature of these compounds to human health have led to numerous studies about the degradation pathways and natural attenuation in subsurface environments [1,2,5,6,7]. The most prevalent biodegradation of CAHs is reductive dechlorination, in which perchloroethene (PCE) converts to trichloroethene (TCE) to dichloroethene (DCE) to vinyl chloride (VC) to ethene. Under aerobic and some anaerobic conditions, DCE and VC can facilitate microorganisms by serving as primary substrates and be degraded [8,9]. Some research also indicated that the degradation of TCE, DCE, and VC are susceptible to co-metabolism [10]. With the biodegradation of PCE and TCE, the daughter products DCE and VC may accumulate and result in severe health problems since VC is more toxic [7,8,9,11]. Much research was centered on the degradation of highly chlorinated hydrocarbons [7,8,9,11] and, to provide more knowledge about the natural degradation of DCE in groundwater, this study focused on the natural degradation of DCE in a pesticide-contaminated site.

The first-order kinetics can be used to model natural attenuation, and it is common to assume that this is appropriate at many sites [8,9]. However, the first-order kinetics do not account for the limitation of contaminant degradation rates by the presence of some compounds other than the contaminant [8]. Recently, advances in high-performance computing and developments in computer sciences have caused a growing interest in using deep learning methods for prediction [12,13]. Deep learning methods have proven to be a powerful tool for forecasting, due to the capacity of the methods to learn shared uncertainties and learn from long-term patterns for both linear and non-linear problems [12,13]. Considering that in the natural environment, complex kinetics take place and there are various underlying interactions between different compounds, the deep learning method has been used to forecast the degradation of DCE in this study.

In this paper, long short-term memory (LSTM) and extreme gradient boosting (XGBoost) were adopted to forecast the degradation of DCE in the contaminated site. LSTM showed excellent performance in predicting long-term hydrological or hydrometeorological time-series and water quality in lakes [14,15,16,17]. XGBoost, as an ensemble model, makes estimations using the average of a large number of simple models and is capable of capturing complex information of data. It has been widely used in water quality predictions [18,19,20]. However, few studies about these two deep learning methods focused on predicting the natural degradation of DCE in groundwater. To learn about how the variables influenced prediction results, SHapley Additive exPlanations (SHAP) analysis was conducted in this study. SHAP are capable of yielding feature impacts of every single instance and bringing the guaranteed explanations of them [21,22]. To the best of the authors’ knowledge, this is the first case where the deep learning methods and SHAP analysis have been used in predicting the natural degradation of DCE.

In this work, a former pesticide manufacturing plant co-contaminated with CAHs and BTEX was selected for the extended monitoring of natural degradation for 5 years. The most crucial biodegradation process for CAHs is reductive dechlorination, and the changes in some indicators are closely related to the process, such as oxygen (DO), pH, and oxidation–reduction potential (ORP) [8]. In this regard, these indicators were used to train the model. The objectives of this work are to (i) use, for the first time in natural attenuation forecasting, deep learning models to forecast DCE concentration; (ii) compare forecasting performance between two deep learning methods; (iii) evaluate the influence of environmental characteristics on a model’s predictive performance using SHAP analysis. This study aims to provide a scientific basis for effectively forecasting the natural attenuation of DCE in contamination sites using the deep learning methods and connecting environment variables with DCE prediction by the aid of SHAP analysis.

## 2. Materials and Methods

### 2.1. Study Site

This work was carried out in a closed pesticide plant in China which had a production history from 1958 to 2006. The main products were pesticides and other chemical materials. The soil between 0 and 6 m underground was treated with ex situ thermal desorption remediation before the monitoring began, however, high levels of pollutants could still be detected in the groundwater.

**Figure 1:**
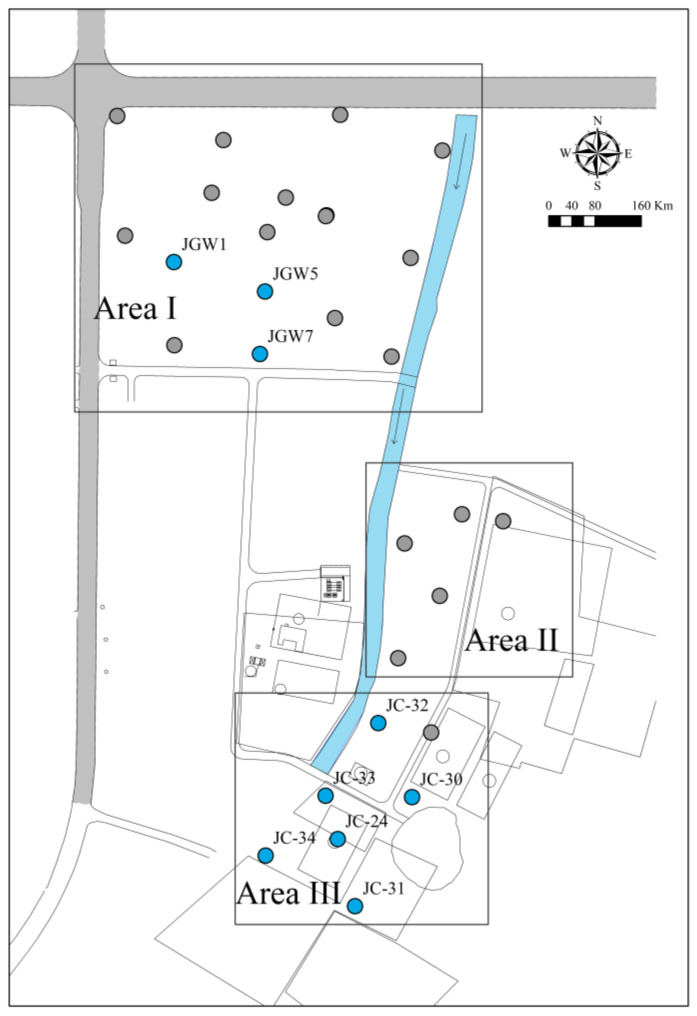
The layout of monitoring wells in the study site. (The gray points are monitoring wells and the blue points are the wells with high levels of DCE concentrations).

A total of 28 monitoring wells were installed for monitoring. In each well, samples from the shallow and deep zone in groundwater were collected (around 8 m and 14 m below the ground surface, respectively). Therefore, wells used to collect shallow groundwater were coded as XX-8m, and wells to collect deep groundwater were coded as XX-14m. The layout of groundwater monitoring wells in this site is shown in Figure 1.

This site was mainly polluted by CAHs and BTEX. According to the manufacturing history in each region and their pollution similarities, this site was divided into three main pollution areas. The main pollutants with high detection rates in Area I were benzene, methylbenzene, ethylbenzene, m&p-xylene, o-xylene, chlorobenzene, 1,2-dichloroethane, and cis-1,2-dichloroethene (cis-1,2-DCE), their maximum concentrations were 55.9, 275, 46.4, 38.6, 129, 49.3, 586, and 3.78 mg/L, respectively. The main pollutants in Area II were chlorobenzene, 1,4-dichlorobenzene, 1,2-dichlorobenzene, methylbenzene, 2-chlorophenol, and benzene, their maximum concentrations were 9.2, 1.81, 1.87, 36.5, 62.1, and 4.69 mg/L, respectively. The main pollutants in Area III were 1,1-dichloroethene (1, 1-DCE), chloroethylene, 1,1-dichloroethane, trans-1,2-dichloroethene (trans-1,2-DCE), 1,2-dichloroethane, trichloroethylene, 1,1,2-trichloroethane, and cis-1,2-dichloroethene (cis-1,2-DCE) and their maximum concentrations were 466, 214, 398, 136, 10.4, 388, 1250, and 33.4 mg/L, respectively.

### 2.2. Sampling Method

Low flow purging and sampling equipment (MP50, QED Environmental Systems, Coventry, UK) was used to conduct the stratified sampling of groundwater. The flow velocity of pumping water was stable and from 100 to 500 mL/min. During the pumping process, temperature, pH, ORP, DO, and conductivity were measured until stable, and the sampling process was finished. The volatile organic compound (VOC) analysis samples were then collected in 40 mL brown glass bottles with hydrochloric acid (1 mol/L) to inhibit the degradation. The sampling interval was every 2–3 months from 2016 to 2021.

### 2.3. Laboratory Analysis

The temperature, pH, ORP, DO, and conductivity were measured by the water quality multi-parameter flow cell 71,790 during the sampling process. The VOCs were analyzed by SEP Analytical Technology (Shanghai) Co., Ltd. (Shanghai, China) following the standard of Method 8260C: Volatile organic compounds by gas chromatography/mass spectrometry. The VOC analysis was conducted using a purge and trap concentrator (Eclipse 4552&4660, OI Analytical, College Station, TX, USA) coupled to a gas chromatography–mass spectrometry system (7890B/5977B, Agilent, Santa Clara, CA, USA) equipped with a capillary column (J&W Scientific (Folsom, CA, USA) DB-624 60 m × 0.25 mm × 1.4 μm, Agilent). The quantitation limits of the pollutants are provided in Supplementary Table S1.

### 2.4. Prediction Model

#### 2.4.1. Variables’ Selection

In order to forecast the concentration of DCE, cis-1,2-DCE and 1,1-DCE were selected as examples. According to the pollution characteristics, the main DCE in Area I was cis-1,2-DCE (mean concentration: 316 μg/L), and in Area III it was 1,1-DCE (mean concentration: 9859.16 μg/L). The shallow and deep wells of JGW1, JGW5, and JGW 7 in Area I were selected to forecast cis-1,2-DCE, and JC24, JC30, JC31, JC32, JC33, and JC34 in Area III were selected to forecast 1,1-DCE. In Area II, DCE concentrations were not at a high level (mean concentration of cis-1,2-DCE: 57.2 μg/L; mean concentration of 1,1-DCE: 109.27 μg/L). No wells in this area were selected.

Geochemical parameters were used to characterize the biodegradation process in natural attenuation, including pH, DO, ORP, and temperature [8]. Some organic compounds could provide carbon and energy sources for chloride reduction, including BTEX, TOC, and other daughter products of PCE and TCE. In view of the practical sampling indicators in the site, there were 11 variables selected as input variables, which were BTEX (benzene, methylbenzene, ethylbenzene, m- and p-xylene, and o-xylene), vinyl chloride (VC), and 5 water quantity indicators (pH, DO, temperature, ORP, and conductivity). Eleven variables were sampled in each well, but some of them had concentrations below the detection limits and therefore were not input into predictive models. Variables with low concentration were considered weakly related to DCE and were not supposed to impact the model’s predictive performance, and herein the number of input variables for each well was different.

For JGW-X, the input variables were BTEX, VC, and 5 water quantity indicators. For JC-X, the input variables were not the same, but they all had 5 water quality indicators at least. In JC31, JC33, and JC30-8m, the input variables were only water quality indicators. In JC24 and JC34, the input variables included some indicators of BTEX, VC, and water quality indicators. Meanwhile, in JC32 and JC30-14m, the input variables included VC and water quality indicators. Input variables of each well are listed in Supplementary Table S2.

#### 2.4.2. Data Processing Method

A data processing method was performed for the dataset to handle missing values and different sampling intervals. Since the sampling interval was around 2 or 3 months and to make the time-series dataset evenly distributed, a resampling method with intervals of 3 months and the linear interpolation were adopted. Moreover, in order to ensure that the model could achieve the forecasting, the model was trained with sequential data which means the data needed to be arranged in chronological order. Data were scaled by the min–max scaler before being inputted into the model to make them converge fast [14]. The data processes were handled in Python 3.7.

#### 2.4.3. Model Description

##### Long Short-Term Memory (LSTM)

**Figure 2:**
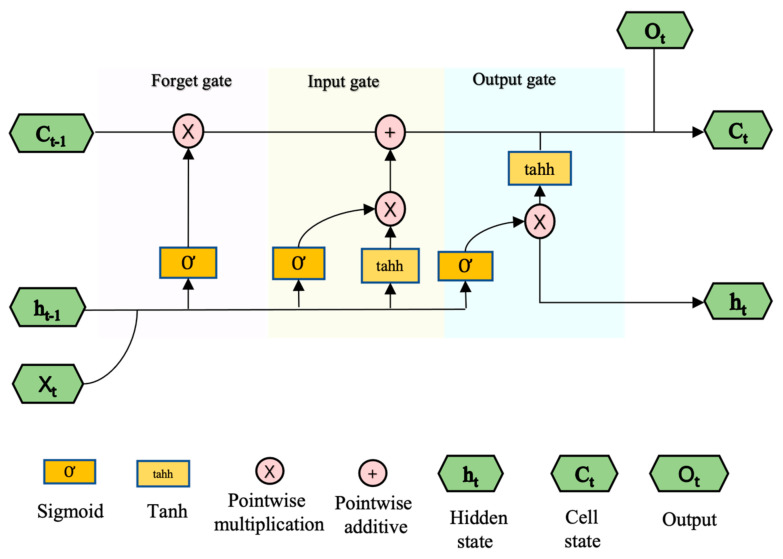
Flow chart of a block in a long short-term memory (LSTM) neural network.

The long short-term memory neural networks were first proposed by Hochreiter and Schmidhuberin [23] to overcome the limitations (gradient vanishing and the exploding gradient problem) of recurrent neural networks (RNNs) when predicting long-term sequential data. The main objective is to allow LSTM to learn long-term dependencies and save information for prolonged periods. The LSTM has a self-connection mechanism controlled by a multiplication gate that learns and decides when to clear the memory content by another unit [24]. The structure of the LSTM neural network is shown in Figure 2, which comprises different memory blocks called cells. The cell has three gates to learn and decide when to forget: the input gate, the output gate, and the forget gate. The forget gate is the first gate encountered by data, and it decides how much of the information should be discarded and it forgets the previous dependence and focuses only on the newer dependence. The second gate is the input gate which decides what and how much information to remember. The output gate decides the output information in the current state. With the function of the three gates, the LSTM model can update the cell unit at each time and learn the long period trend. Detailed information about LSTM is presented in Supplementary Text S1.

For LSTM analysis, we used lstm in tensorflow.keras 2.2.0 of Python 3.7.

##### Extreme Gradient Boosting (XGBoost)

XGBoost is an ensemble method with many weaker models, as opposed to being a single, highly complex model (i.e., LSTM, RNN) [20]. Ensembles are constructed from many decision tree models. Like a tree model, it splits data according to features. Trees are added each time to fit the model in order to correct the prediction errors made by prior models. Each leaf in a tree represents a numerical weight, and each sample is assigned to a set of leaves based on the values of its input variables. The model’s estimated output for that sample is obtained by adding the sum of the leaves assigned to that sample for each regression tree [18]. Detailed information about XGBoost is presented in Supplementary Text S2.

For XGBoost analysis, we used the Python package XGBoost 1.6.0.

##### SHapley Additive exPlanations (SHAP)

SHAP analysis was used to evaluate variables’ importance to models. SHAP analysis is a locally accurate and consistent feature attribution method that provides more stable rankings than previous importance measures [21,22]. SHAP values attribute the marginal contribution from each predictor variable to each prediction (measured relative to the average prediction).

The formula for SHAP values is as in Equation (1) [21]:

(1) $$\psi_{i}=\underset{S\subseteq F\backslash \left\{i\right\}}{\sum }\frac{\left|S\right|!\left(F-\left|S\right|-1\right)!}{\left|F\right|!}\left(f\left(S\cup \left\{i\right\}\right)-f\left(S\right)\right)$$

where *F* is the total number of input features and *S* is subsets of *F* without feature *i*. $\psi_{i}$ is the additive feature attribution about the feature *i* which is also called the SHAP value. $f\left(S\cup \left\{i\right\}\right)$ is a model trained with feature *S* and *i*, while $f\left(S\right)$ is a model trained with feature *S*. The effect of feature *i* is evaluated by the difference between $f\left(S\cup \left\{i\right\}\right)$ and $f\left(S\right)$. The larger the absolute SHAP value for feature *i* is, the more important this indicator is. The positive SHAP values indicated that the feature had a positive impact on models’ output, and negative values indicate a negative effect on models’ output. For SHAP analysis, we used the Python package shap 0.40.0.

#### 2.4.4. Model Training and Evaluation

##### Model Training

To acquire a good prediction, the parameters in LSTM were analyzed, and finally an optimal parameter: a batch size of 15, epochs of 80 and nodes of 50, and activation function of RELU were used for each well.

As for XGBoost, the optimal three hyperparameters (max_depth, learning_rate, n_estimators) for each well were found by GridSearchCV (max_depth, 5, 10, 15, 20; learning_rate, 0.01, 0.05, 0.1, 0.15, 0,2, 0,3; n_estimators, 50, 100, 200, 300, 500, 800, 1000), and the best three parameters are shown in Supplementary Table S3.

The training instance length was 3 months which means variables of the previous 3 months were used for training and forecasting the DCE concentrations. Since data were resampled to a 3-month interval, the input variables in models were data of one sampling, and the prediction was the prediction of the next sampling. The data were split into the training period (1 July 2016 to 31 December 2019) and testing period (1 January 2020 to 31 July 2021, 6 sampling data), corresponding to a total of 67% and 33% data for training and testing. Since the sampling data size of each well was slightly different, to make the prediction data size equal, we fixed the same testing data size to be 6 for each well. Previous data were used for training, but the training data size was also different for each well. This could lead to data disparity and varying spatial coverage in different wells.

##### Model Evaluation

The predictive concentrations were evaluated against the concentration analyzed in the laboratory. To assess the performance of the proposed method, three of the most widely used evaluation metrics were adopted: the mean absolute error (MAE), mean absolute percentage error (MAPE), and root mean squared error (RMSE).

(2) MAE=∑i=1nyi^−yin

(3)   MAPE=1n∑i=1nyi^−yiyi

(4) RMSE=∑i=1nyi^−yi2n

where yi^ and $y_{i}$ are the predictive and actual values of the observation *i* = 1, 2, 3…*n*, and *n* is the total number of observations.

## 3. Results

### 3.1. Characteristics of Selected Wells

**Figure 3:**
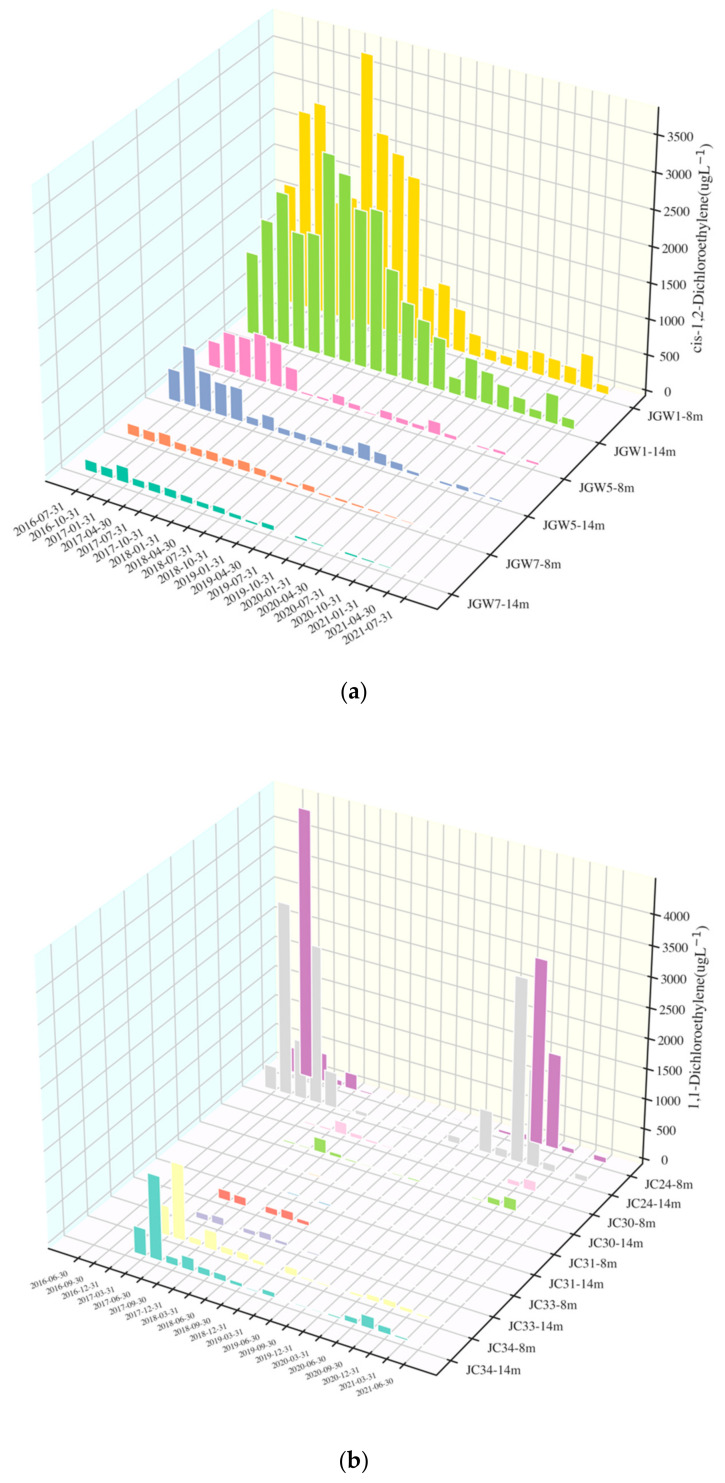
Concentration variation with time of cis-1,2-DCE and 1,1-DCE. ((**a**) Wells with cis-1,2-DCE, (**b**) wells with 1,1-DCE. JC32 is not shown since the magnitude of its concentration was larger than that of other wells).

The characteristics and descending trend of selected wells are listed in Table 1, and the concentration variation with time is shown in Figure 3. The detailed characteristics of variables in each selected well are listed in Supplementary Table S4. Statistical trend analyses of DCE were calculated by the Mann–Kendall test. The negative tau-values represented a descending trend, while the positive value represented an increasing trend. For the *p*-value, a value smaller than 0.05 meant a significant descending trend, and an apparent descending trend showed the high possibility of natural attenuation happening in the well.

Cis-1,2-DCE was mainly detected in JGW1, JGW5, and JGW7. In JGW1-8m, the concentration of cis-1,2-DCE ranged from 127–3780 μg/L, with an average of 1273.33 μg/L, which was the highest concentration among other wells (mean concentrations of cis-1,2-DCE in JGW1-14, JGW5-8m, JGW5-14m, JGW7-8m, JGW7-14m were 1185.95 μg/L, 194.27 μg/L, 204.75 μg/L, 78.16 μg/L, 75.08 μg/L, respectively). A significant variation in cis-1,2-DCE over time was also found in JGW1-8m. The concentration of cis-1,2-DCE in all wells had a significant descending trend, with negative tau-values and *p*-values smaller than 0.05. The concentration of cis-1,2-DCE in JGW7-8m had the most surprising descending trend with a tau-value of −0.87.

1,1-DCE mainly existed in JC24, JC30, JC31, JC32, JC33, and JC34. The deep well of JC32 had a concentration of 1,1-DCE ranging from 36.9–115,000 μg/L, with an average of 27,074.67 μg/L, which was a highly polluted well (mean concentrations of 1,1-DCE in JC24-14m, JC24-8m, JC30-14m, JC30-8m, JC31-14m, JC31-8m, JC32-8m, JC33-14m, JC33-8m, JC34-14m, JC34-8m were 662.29 μg/L, 516.47 μg/L, 53.03 μg/L, 48.30 μg/L, 9.45 μg/L, 6.13 μg/L, 20,623.70 μg/L, 35.59 μg/L, 43.19 μg/L, 177.02 μg/L, 170.98 μg/L, respectively). The variation in 1,1-DCE concentration over time in JC32-14m was obvious among other wells. An evident descending trend was found in JC32-14m, with a tau-value of −0.7 and a *p*-value of 1.18 × 10^−5^. Most wells had significant negative tau-values with *p*-values less than 0.05, except for JC24 and JC30. In JC24 and JC30, the concentration of 1,1-DCE reached a peak during the year 2017 and then decreased, while it had an increase during August and November 2020, which was contrary to the descending trend. A sharp increase in concentrations was also found by Rahim et al. [6], which is related to the rain intensity. The high rain intensity elevated the groundwater table, which would then bring 1,1-DCE back to the groundwater from soil or sand [25,26,27].

Overall, the highest concentrations of 1,1-DCE were larger than that of cis-1,2-DCE, while the descending trend of 1,1-DCE was not as significant as cis-1,2-DCE on the whole.

### 3.2. Prediction Results of XGBoost and LSTM

**Figure 4:**
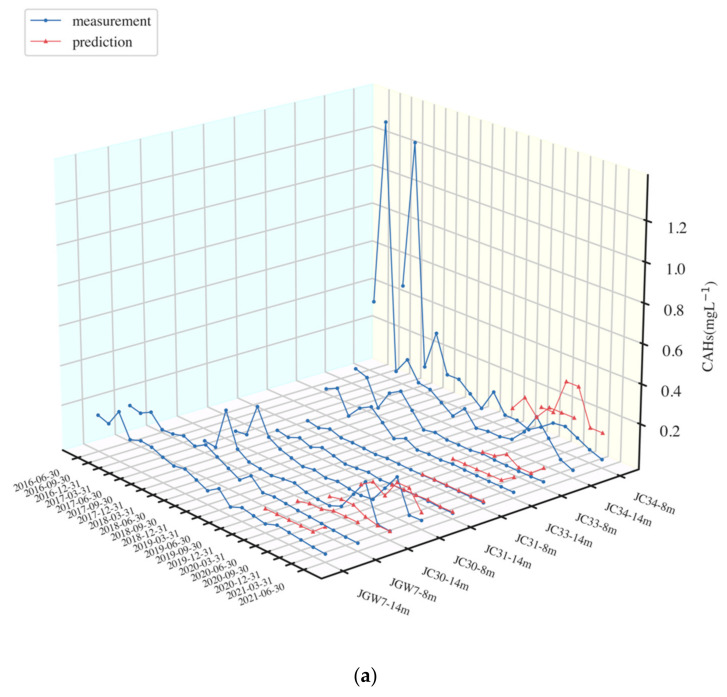

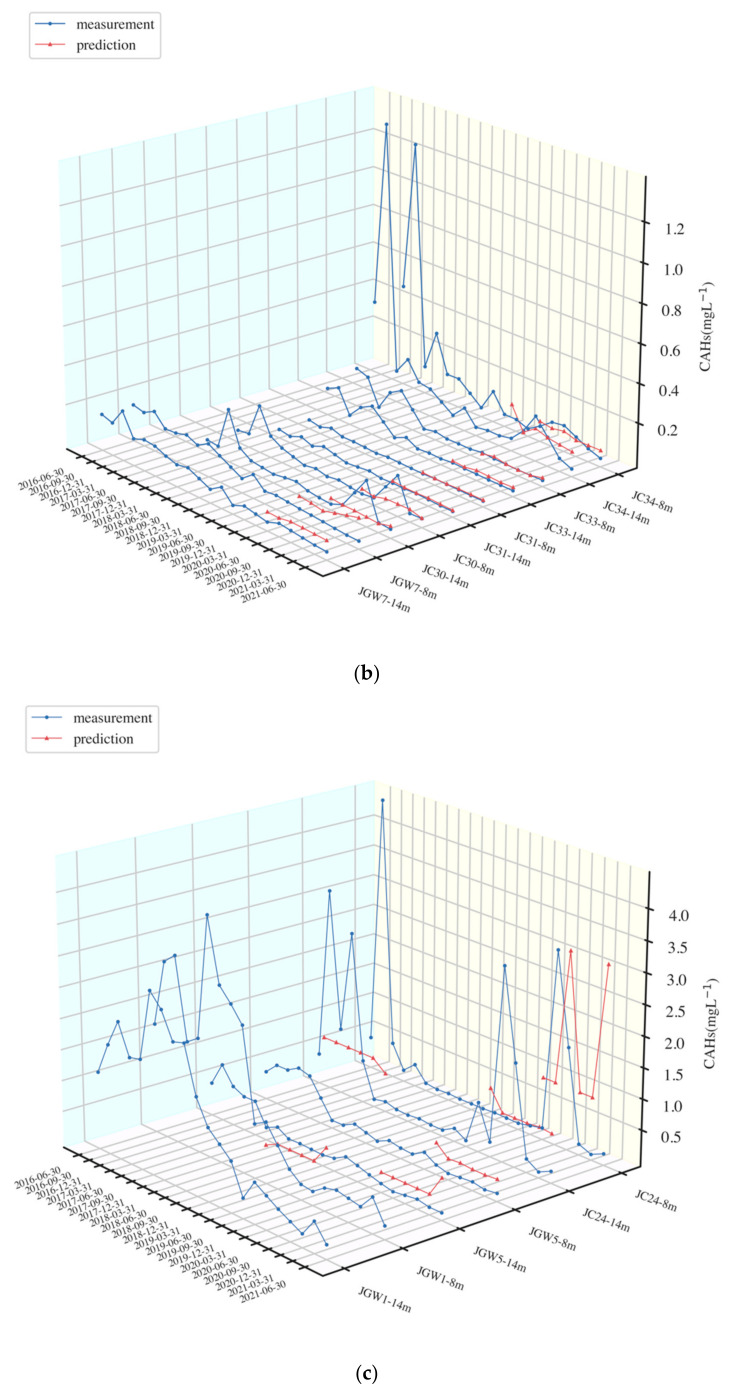

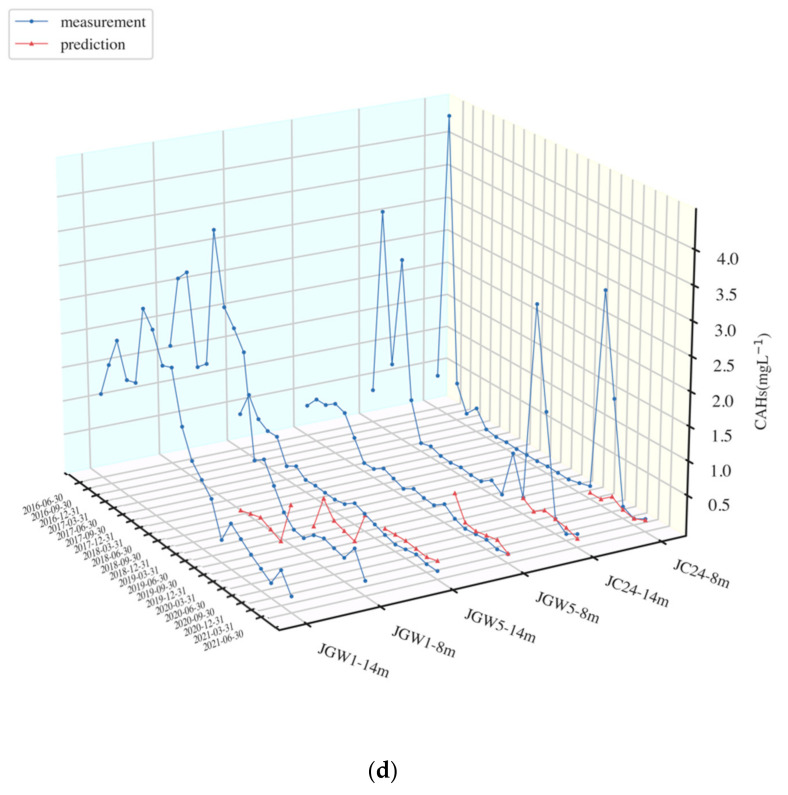
Prediction results against the measurement of XGBoost and LSTM (mg/L) ((**a**) wells with a small prediction error of XGBoost; (**b**) wells with a small prediction error of LSTM; (**c**) wells with a relatively large prediction error of XGBoost; (**d**) wells with a relatively large prediction error of LSTM. The prediction results of JC32-8,14m are shown in Supplementary Figure S1).

The predictive performances of XGBoost and LSTM for cis-1,2-DCE and 1,1-DCE were evaluated in terms of RMSE, MAE, and MAPE, and the results are presented in Table 2. Predictive results against lab measurements of two models are presented in Figure 4 and Supplementary Figure S1.

In general, the LSTM model showed a better performance in predicting cis-1,2-DCE and 1,1-DCE. For cis-1,2-DCE, the average RMSE, MAE, and MAPE of LSTM were 0.28, 0.24, and 3.6, respectively. The results of the LSTM model showed a reduction of 58.47%, 63.86%, and 48.33% on average RMSE, MAE, and MAPE compared to XGBoost. For 1,1-DCE, the average RMSE, MAE, and MAPE of LSTM were 3.13, 2.52, and 32.05, respectively. The results of the LSTM model showed a reduction of 60.98%, 67.23%, and 65.61% on average RMSE, MAE, and MAPE compared to XGBoost.

For individual wells, the RMSE value of the LSTM model was smaller than that of XGBoost, except for the wells JC24-14m, JC30-14m, and JC31-14m. The difference in RMSE, MAE, and MAPE between the two models in JC30-14m and JC31-14m was minor, while the difference in JC24-14m was larger. In JC24-14m, the RMSE of LSTM was larger than that of XGBoost, while the MAE and MAPE of LSTM were much smaller than XGBoost. The reason is that testing instances with rather small errors would contribute to the low MAE and MAPE values, while test instances with rather large errors would result in high RMSE values. RMSE would amplify the large errors [28].

In Figure 4a,b, the evaluation metrics of XGBoost and LSTM models were small, while they were relatively large in Figure 4c,d. XGBoost and LSTM models showed a similar pattern of overestimating the measurement. However, the XGBoost had larger biases in most wells than LSTM. For example, in JC24, the range of prediction results of XGBoost (0 to 3 mg/L) was larger than that of LSTM (0 to 0.1 mg/L).

In the shallow and deep wells of JC30, the measurements of data in the testing period were not steady, which increased first but then decreased, and this may be caused by rain intensity, as mentioned before. XGBoost performed better than LSTM to capture this unsteady trend (Figure 4a,b), and the evaluation metrics of XGBoost were smaller than that of LSTM in JC30-14m. However, in JGW1, JC24, and JC34, the prediction results of XGBoost were largely affected by its previous trend, which resulted in large biases than measurement. By contrast, LSTM showed a steady prediction, but it was hard to predict the maximum value. The same difference between LSTM and XGBoost was also found in the research by Cerna et al. [29].

It seems like XGBoost was more likely to capture the variation in DCE concentrations and be robust in high values than LSTM. However, the LSTM model presented better accuracies for all the wells, had a steady trend or peak trend of data in the testing period, and its evaluation metrics surpassed those from XGBoost.

### 3.3. SHAP Analysis on XGBoost and LSTM

SHAP values were used to evaluate variable importance and behavior. To acquire them, SHAP analysis was conducted on XGBoost and LSTM models. The mean absolute SHAP value of each variable was scaled by the sum of them, and the results of each well are shown in Supplementary Figures S2 and S3.

There were 11 variables in each well of JGWX. For XGBoost, a mean absolute SHAP value larger than 0.5 was found in m- and p-xylene, DO, and ORP, and the less important variables were methylbenzene, o-xylene, and pH with a mean absolute SHAP value larger than 0.3. For LSTM, no variable had a mean absolute SHAP value larger than 0.5, and each variable in one well had a relatively uniform mean absolute SHAP value compared to XGBoost. The most important variables were ORP, ORP, methylbenzene, ethylbenzene, methylbenzene, and benzene in JGW7-14m, JGW7-8m, JGW5-14m, JGW5-8m, JGW1-14m, and JGW1-8m, respectively. In general, BTEX, DO, and ORP had significant influences on the two models in predicting cis-1,2-DCE concentrations.

In JCX, wells had different input variables, but all of them had five water quality indicators at least. In terms of XGBoost, conductivity, pH, and DO had large mean absolute SHAP values in most wells, except for JC24-8m, which was dominated solely by benzene. VC was also important to JC32 and JC34-14m. However, BTEX did not contribute a lot to the model’s prediction of JCX compared to JGWX. As for LSTM, conductivity, temperature, and DO were found to have large mean absolute SHAP values in many wells. Except for water quality variables, VC was also important in some wells. Similar to the trend in JGWX, variables in JCX had mean absolute SHAP values much larger than others in XGBoost, while each variable showed a relatively small different mean absolute SHAP value in LSTM.

In general, each variable had relatively equal importance in the LSTM model, while the XGBoost had predominate variables which had large mean absolute SHAP values. BTEX had large influences on models’ output in JGWX, while they had comparatively small impacts on models’ output in JCX, which were largely affected by VC. Water quality indicators such as ORP and conductivity played an essential role in prediction.

### 3.4. Prediction Results of Water Quality Indicators

**Figure 5:**
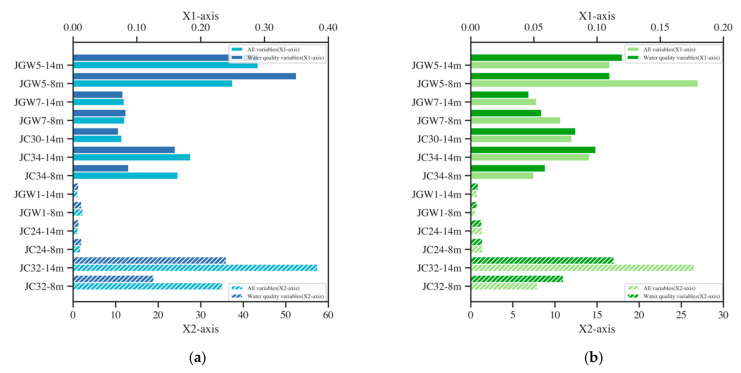
Comparison of RMSE between models ((**a**) XGBoost and (**b**) LSTM) with all variables and water quality variables only (JC30-8m, JC31-8,14m, and JC33-8,14m were removed from all wells since they did not have organic variables).

With regard to the importance of water quality variables to models’ prediction and the cost-effectiveness of acquiring them, five water quality variables were used to predict the concentration of cis-1,2-DCE and 1,1-DCE by LSTM and XGBoost. The RMSE of the prediction was compared with that using all variables (including BTEX and VC), and the result is shown in Figure 5.

**Figure 6:**
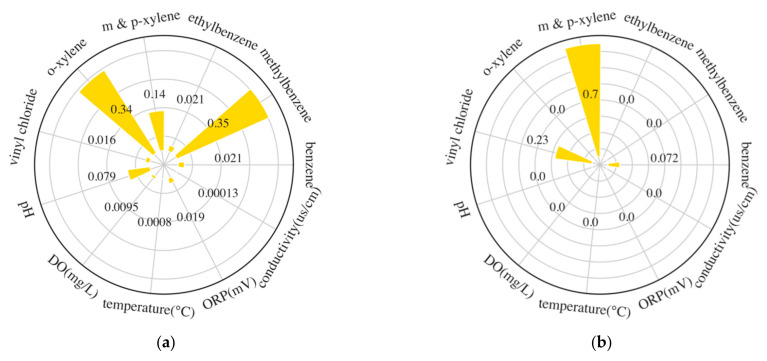

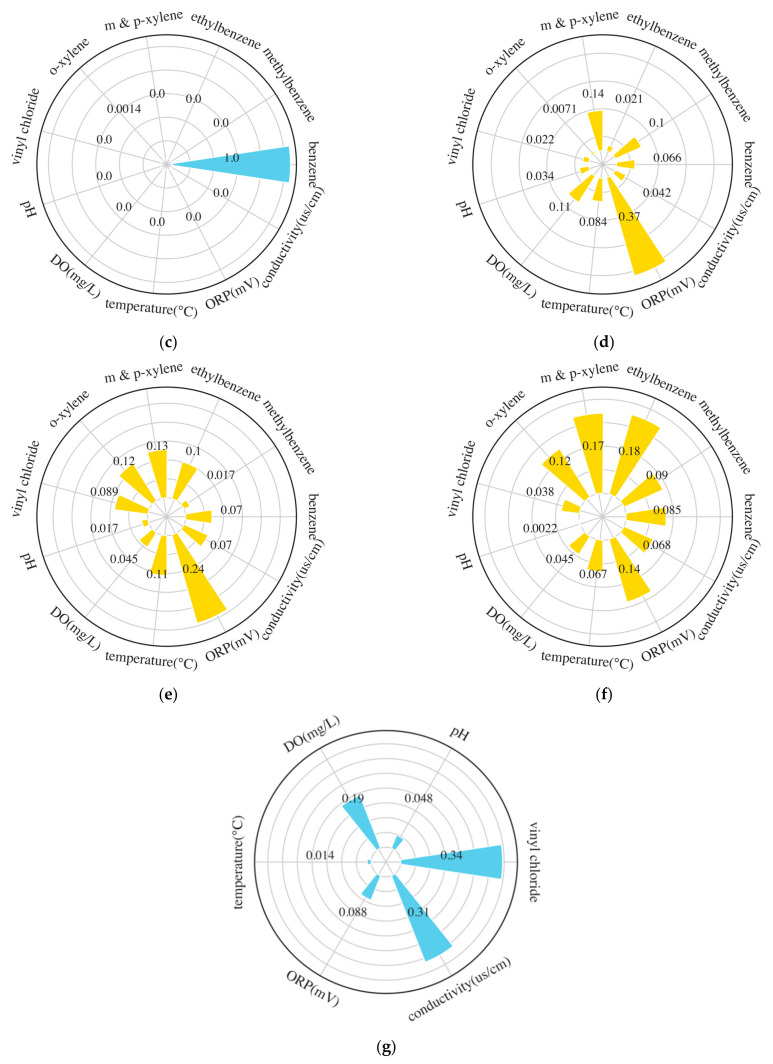
Mean absolute SHAP value of each variable in some wells. (**a**) JGW1-14m (XGBoost); (**b**) JGW5-14m (XGBoost); (**c**) JC24-8m (XGBoost); (**d**) JGW7-14m (LSTM); (**e**) JGW7-8m (LSTM); (**f**) JGW5-8m (LSTM); (**g**) JC32-14m (LSTM).

XGBoost performed poorly in JGW1-14m, JGW5-8m, and JC24-8m since the RMSE of XGBoost with input water quality variables was larger than that of all variables. The mean absolute SHAP values of water quality variables in them were nearly or equal to 0 (Figure 6a–c), therefore water quality variables could hardly provide enough information for predicting, which then resulted in bad prediction. In JC32-8,14m and JC34-8,14m, models with water quality variables performed better than models with all variables. The mean absolute SHAP value of VC or benzene in JC32-8,14m and JC34-8,14m was found to be larger than 0.4. However, DO also showed relatively high importance. Therefore, after removing these organic variables, models could still perform well with water quality variables. For other wells, the RMSE between the prediction result of XGBoost with water quality indicators and all variables showed an insignificant difference since most wells had water quality variables as the most important variables and therefore DCE was forecast without organic variables.

For the LSTM model, the prediction errors in JGW5-8m, JGW7-8,14m, and JC32-14m of the model with water quality variables were more minor than those with all variables. In JGW7-8,14m, ORP was the predominant variable, while the organic variable showed relatively little importance to models (Figure 6d,e). Some research manifested that unnecessary input variables may be deleterious to model performance [30]. Therefore, eliminating these variables can improve model performance. In JGW5-8m and JC32-14m (Figure 6f,g), although organic variables (BTEX, VC) shared similar importance as ORP or conductivity, after removing them, models could also be improved since they could learn from some variables which were highly correlated to the organic variables.

In general, for wells with water quality variables of high SHAP value, removing insignificant important organic variables could improve models’ performance. However, if water quality variables had little importance, removing organic variables would result in a bad prediction. The results indicated that LSTM and XGBoost could predict DCE concentrations with only water quality variables, since for most wells, the differences in RMSE between the models with water quality variables and with all variables were relatively small. LSTM with water quality indicators performed better than XGBoost in forecasting DCE concentrations.

## 4. Discussion

### 4.1. Influences on Models’ Prediction

#### 4.1.1. Influences of DCE Concentrations

**Figure 7:**
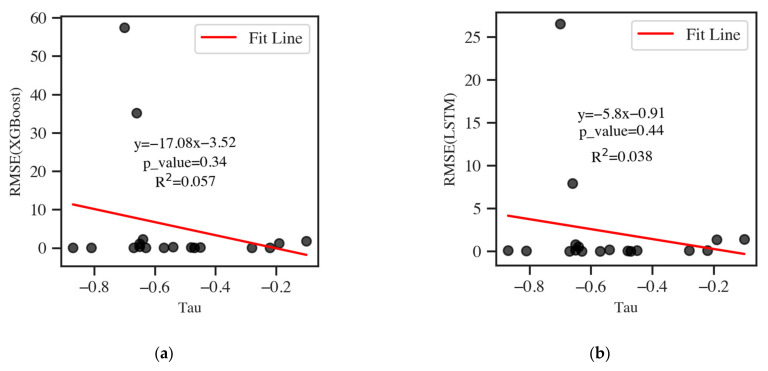

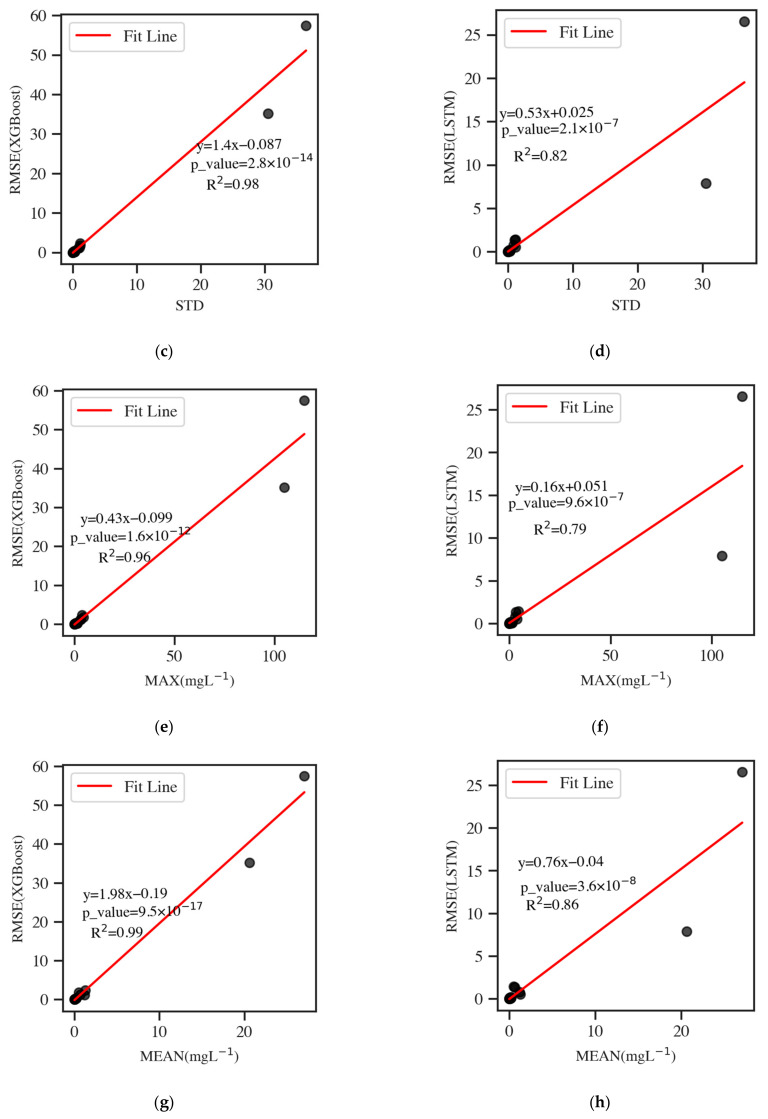

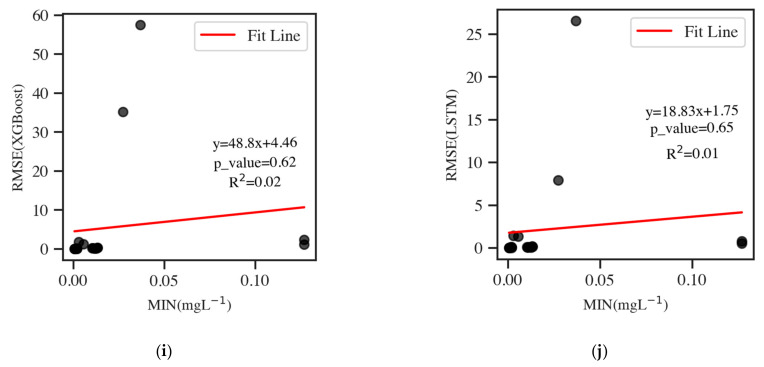
The RMSE of two models ((**a**,**c**,**e**,**g**,**i**) XGBoost and (**b**,**d**,**f**,**h**,**j**) LSTM) against tau-values (descending trend calculated by Mann–Kendall), maximum, mean, and standard deviation of DCE concentrations.

The RMSE of XGBoost and LSTM against the descending trend, and the standard deviation, mean, maximum, and minimum of DCE (cis-1,2-DCE or 1,1-DCE) concentrations are presented in Figure 7.

The RMSE of XGBoost and LSTM decreased with the tau-values, but R^2^ values and *p*-values of fitted lines indicated an insignificant trend. Therefore, the models’ predictive performance had a weak correlation with the descending trend.

The standard deviation of DCE showed the variation in concentration, and the RMSE values of the two models were positively correlated with it. The R^2^ values of fitted lines were 0.98 and 0.82 in XGBoost and LSTM, respectively, and the *p*-values of them were smaller than 0.01. The result showed RMSE values significantly positively correlated with variation in DCE concentration. This indicated that wells with small DCE variations had a low error and better predictive performance.

The RMSE values of the two models were positively correlated to the maximum and mean concentration of DCE. The R^2^ values of XGBoost were 0.96 and 0.99, and the values of LSTM were 0.79 and 0.86, respectively. The *p*-values of them were smaller than 0.01, which showed RMSE values significantly positively correlated with maximum and mean of DCE concentrations. The R^2^ value of RMSE against the minimum concentration of DCE of the XGBoost and LSTM did not show the same significant positive correlation with R^2^ values of 0.02 and 0.01, respectively, which manifested that the influence of the minimum concentration of DCE was hardly comparable to that of the maximum concentration of DCE. The result indicated that wells with higher DCE concentrations would cause a higher error in the model’s accuracy. The influences of DCE concentrations on XGBoost were more significant than on LSTM because the slope of the fitted line of XGBoost was larger than that of LSTM. This finding corresponded to the result of LSTM performing better than XGBoost with small evaluation metrics.

#### 4.1.2. Influences of Variables

##### Water Quality Indicators

**Figure 8:**
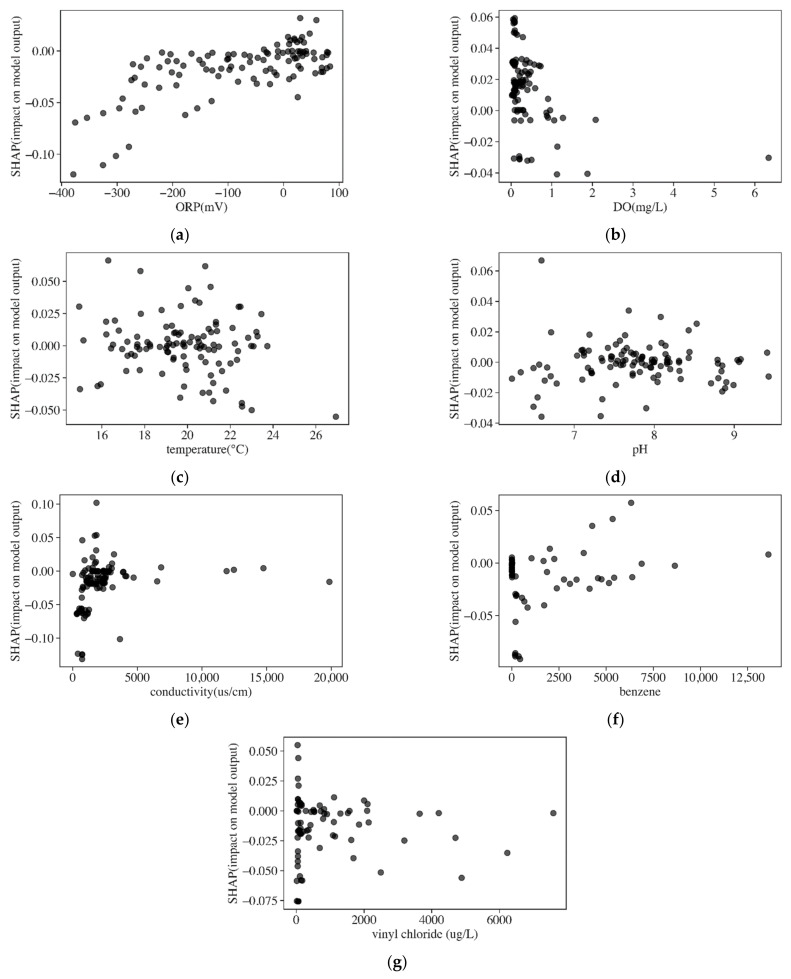
The SHAP value dependent plots of water quality indicators, benzene (µg/L), and VC (µg/L). (**a**) ORP, (**b**) DO, (**c**) temperature, (**d**) pH, (**e**) conductivity, (**f**) benzene and (**g**) vinyl chlo-ride.

**Figure 9:**
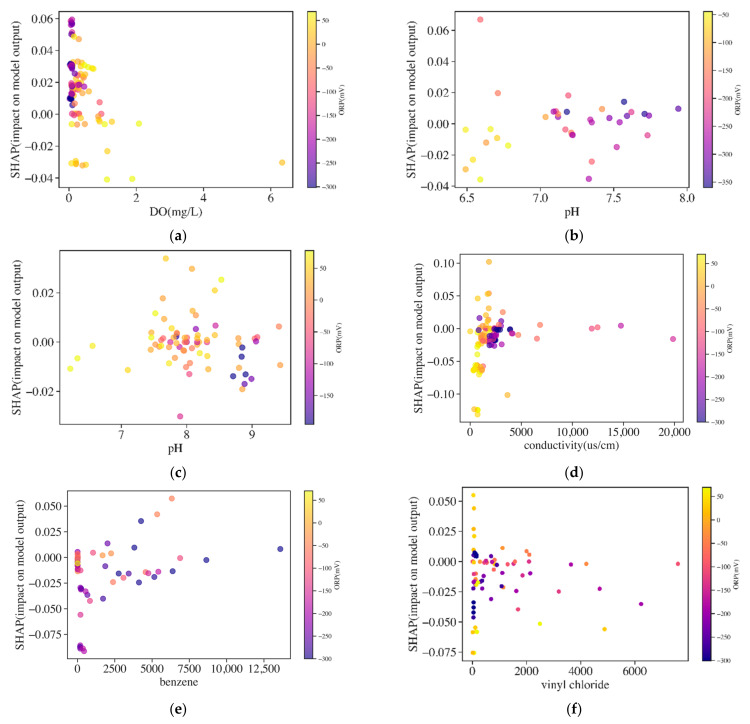
The SHAP value dependent plots of (**a**) DO, (**b**) low pH, (**c**) high pH, (**d**) conductivity, (**e**) benzene (µg/L) and (**f**) vinyl chloride (µg/L) colored by ORP values.

The general impact of each variable was shown by the mean absolute SHAP value. To know how the variables of each instance (data) impacted models’ prediction, detailed SHAP values of each instance against corresponding variables’ values were analyzed. The SHAP dependent plots of five water quality variables, benzene, and VC in LSTM are shown in Figure 8. The positive SHAP values indicated that variables had positive impacts on predictions, and negative values indicate negative impacts. The SHAP dependent plots of DO, pH, conductivity, and benzene are colored by ORP values to show the influences of ORP on them in Figure 9.

ORP measures the electron activity and the relative tendency for the solution to transfer or accept electrons. Negative ORP values indicated the existence of anaerobic conditions in the groundwater and the suitable condition for reductive dechlorination was when ORP was less than 50 mV [8]. In Figure 8a, ORP values ranged from −400 mV to 100 mV, and negative SHAP values increased with ORP values until ORP values were larger than around 50 mV, and SHAP values became positive. The negative SHAP values of ORP ranging from −400 V to 50 mV indicated the degradation of DCE in the optimal reductive dechlorination condition. In contrast, the positive SHAP values of ORP with concentrations larger than 50 mV indicated the positive impacts on the models’ output, caused by the bad reductive dechlorination condition.

DO is the most thermodynamically favored electron acceptor used by microbes for the biodegradation of DCE under anaerobic conditions [8,31]. Anaerobic bacteria generally cannot function at DO concentrations greater than about 0.5 mg/L, hence, reductive dechlorination will not occur [8]. From Figure 8b, the concentration of DO ranged from 0–6 mg/L and was mainly in the range of 0–1 mg/L. The trend line of DO formed by the scatter points had a dispersion along the vertical direction when DO ranged from 0–0.5 mg/L, which showed that most instances have positive influences on the models’ output. Although DO in the range of 0–0.5 mg/L benefited DCE degradation, the SHAP values did not show the same result. In Figure 9a, ORP contents increased with DO contents. ORP was negative when DO ranged from 0–0.5 mg/L, while in this optimal biodegradation condition, the positive SHAP value of DO indicated the increase in DCE. One explanation for this may be related to the existence of PCE and TCE, which were more likely than DCE to undergo reductive reactions and form the accumulation of DCE. When DO was higher than 1 mg/L, instances had negative SHAP values indicating the degradation of DCE. In this environment, DCE can be utilized as a primary substrate and oxidized under aerobic conditions, therefore DCE would decrease [8].

Groundwater temperature directly affects microbial activity, which readily occurs at 13–26 degrees Celsius (°C) [8]. Some research also confirmed that the degradation of cis-1,1-DCE to VC reached high dechlorination rates at 15–30 °C [32]. The temperature range of 15–26 °C indicated the environment was suitable for biodegradation, while the SHAP values fluctuated up and down at 0. The suitable dechlorination temperature range did not result in a negative SHAP value for all instances, which was related to the complicated reaction that happened in this temperature range, and it indicated that temperature had a relatively small correlation to dechlorination.

The pH of groundwater influences the presence and activity of microorganisms in groundwater. pH values from 6 to 7.5 are optimal for reductive dechlorination of DCE [33,34]. In Figure 8d, pH ranges from 6.5 to 9.5. In the optimal reductive range of pH (6–7.5), most of the instances had negative SHAP values, which corresponded to the degradation in the optimal reductive environment. The positive SHAP value of pH in the range of 7.5–8.5 may be related to the bad conditions for the degradation of DCE. Meanwhile, when pH ranged from 8.5 to 9.5, the SHAP value of each instance was negative which indicated the degradation of DCE but in a bad reductive environment. The SHAP values of pH colored by ORP in cis-1,2-DCE and 1,1-DCE are shown in Figure 9b,c. The low pH would increase ORP, and it was shown that ORP in the low pH was higher than that in high pH in cis-1,2-DCE and 1,1-DCE, respectively. When pH ranged from 7.5 to 8.5, most instances with negative SHAP values had corresponding ORP lower than 0, and the optimal ORP environment may explain the degradation of DCE in the non-ideal pH condition. Meanwhile, in this range, some instances had positive SHAP values with ORP higher than 0. Although pH higher than 8.5 was bad for degradation, most instances had corresponding low ORP (<−50 mV), which benefitted the degradation of DCE.

Conductivity is a measure of the ability of groundwater to conduct electricity. The conductivity of groundwater increases as ion concentration increases [8]. The SHAP values of conductivity were negative and increased with the increase in conductivity until conductivity equaled around 2000 µs/cm, and SHAP values increased to around 0 (Figure 8e). The trend of SHAP values indicated that the negative influences on models became limited when conductivity was higher than 2000 µs/cm. In Figure 9d, ORP decreased with conductivity. ORP was higher than 0 when conductivity was lower than 2000 µs/cm, while ORP was lower than 0 when conductivity was higher than 2000 µs/cm. It was speculated that with the reductive dechlorination process, conductivity continued to increase while ORP would be decreased. During the degradation process, some bacteria may respire using NO_3_^−^, SO_4_, Fe^3+^, or a variety of metals (such as arsenic or uranium) as the oxidant [8]. Each sequential reaction (NO_3_^−^, SO_4_, Fe^3+^, etc.) drives the ORP of the groundwater downward into the range within which reductive dechlorination can occur, and the dechlorination rate would be accelerated with the decrease in redox potential [8]. Therefore, it was supposed that the ORP continued to decrease with the occurrence of reductive dechlorination. In this system, the production of large numbers of inorganic ions such as CL^−^ and Fe^2+^ would increase the conductivity [34].

By comparing the SHAP values’ range of water quality indicators, ORP and conductivity were important variables in prediction. DO, pH, and temperature had a relatively low influence on the models’ performance.

##### Organic Indicators

Organic variables used for forecasting included BTEX and VC. Benzene was selected to be analyzed as the representation of BTEX since it had a large sampling data size and high concentrations, and the SHAP dependent plots of benzene and VC are shown in Figure 8f,g.

The SHAP values of benzene indicated it imposed negative impacts on LSTM. The SHAP value increased to 0 with the increase in benzene until benzene equaled 2500 µg/L. The negative effects on models manifested that it is beneficial to the degradation of DCE. During degradation, BTEX consumed the electron acceptors and released the biodegradable primary substrates to supply the electron donors for dechlorination [8,35]. Therefore, the existence of benzene could support reductive dechlorination and impose negative effects on models’ output. The depletion of electron acceptors would result in decreasing ORP during this process. Figure 9e shows the SHAP value of benzene colored by ORP. Most instances with negative SHAP values had negative ORP, while some instances with SHAP values close to 0 had ORP near to or higher than 0 mV. It was speculated that benzene with a negative SHAP value benefited dechlorination and decreased ORP in the meantime. Similar to conductivity, when benzene increased to a certain concentration, it provided enough primary substrates for reductive dechlorination, and therefore reached a limited influence on the mode, therefore the SHAP value of benzene reached 0 when benzene had concentrations higher than 2500 µg/L.

VC as the degradation product of DCE is formed from cis-1,2-DCE and 1,1-DCE after they receive electrons. The presence of VC indicated the degradation of DCE, and therefore it had a negative impact on the models’ output. The SHAP values of VC mainly exhibited a negative effect, although some data instances had positive SHAP values (Figure 8g). Most instances with negative SHAP values had negative ORP, some instances with positive SHAP values had positive ORP, while other instances with negative SHAP values also had positive ORP (Figure 9f). Most instances having negative ORP indicated a reductive condition and VC as the daughter product revealed the degradation of DCE. For instances with positive ORP but negative SHAP values, some studies found biodegradation of DCE and VC in the aerobic condition and therefore these instances with small VC concentrations showed the degradation of DCE in the relative aerobic environment [9].

In general, the explanation of the SHAP value of each variable indicated the high consistency with the rules of biodegradation in the real environment.

### 4.2. Comparison between XGBoost and LSTM

As mentioned above, XGBoost is an ensemble model consisting of several weaker models to prevent overfitting. Due to this characteristic, it allows each model to capture some aspect of the data structure. Unlike XGBoost, the advantage of LSTM is it stores all the previous steps’ inputs and merges that information with the current step’s input. The ability of LSTM to learn long-term patterns explained its weakness in capturing the variation in testing data compared to XGBoost. In JC24 and 30, although the 1,1-DCE concentration had variation initially, they then went through a relatively steady period until the testing period, which had an increment. XGBoost and LSTM showed significant disparate predictions in JC24 and JC30 as XGBoost had fluctuating predictions while LSTM was steadier.

The XGBoost model depends largely on several variables compared to LSTM. As shown in Supplementary Figures S2 and S3, XGBoost had some variables with SHAP values larger than 0.5, while LSTM seldom had variables with large SHAP values. XGBoost contains several tree models, and each tree model could be split according to features, therefore, if one feature was mostly used or had a high average gain across all splits, it would be important. LSTM had a recurrent structure and different states to remember or forget the new data. The disparate calculations between LSTM and XGBoost explained the different SHAP values of variables in each well.

### 4.3. Suggestions for the Model and the Potential for Low-Cost Modeling

The prediction result of cis-1,2-DCE was better than 1,1-DCE in general. The core pollution area of 1,1-DCE in Area II was at a higher level than cis-1,2-DCE, and the descending trend of cis-1,2-DCE was better than that of 1,1-DCE. The increase in rain intensity elevated the groundwater table, which would then bring 1,1-DCE back to groundwater from soil or sand and then decrease the downward trend. This phenomenon was not presented obviously in cis-1,2-DCE, and since the prediction performance highly correlated to the variation and concentration of DCE, the model’s prediction of cis-1,2-DCE was better than that of 1,1-DCE. In addition, it is possible that the more favorable biodegradation of cis-1,2-DCE than 1,1-DCE contributed to the better prediction performance of cis-1,2-DCE than 1,1-DCE. The greater tendency of cis-1,2-DCE than 1,1-DCE to degrade in certain conditions would contribute to the high correlation between concentrations with water quality variables, which then benefited predictive models learned from them and they performed better. In order to improve the prediction accuracy, some variables related to the rain intensity or groundwater level could be considered as input variables. LSTM had small evaluation metrics compared to XGBoost, especially for testing data with a steady trend. The sharp increment in DCE concentration caused by rain needs to be considered, while if we focus on the natural descending trend in the long term, LSTM performed better.

Considering the lower cost to acquire water quality indicators and the little different prediction performance between models with or without organic variables, water quality variables could be considered for predicting future DCE concentrations, which could reduce the time consumption of traditional CAH concentration analysis methods and certain economic costs. To acquire a more accurate prediction result, it is important to select input variables. In the study, SHAP analysis showed the predominant variables in each well. Therefore, reducing some of the non-significant variables could improve prediction results. For each well, the optimal input combination of variables can be quickly determined by adding the input variables one by one into models according to the order of SHAP values. In Section 3.4, models showed excellent performance in some wells with only water quality indicators. However, other wells performed badly with only water quality indicators. Therefore, for the best use of models, it is suggested to select more important input variables. Meanwhile, the cost and time to analyze these variables should be considered. The findings from reducing some organic input variables manifested the possibility of saving cost by reducing input variables. Therefore, under the premise of not significantly reducing the prediction performance, removing the variables that contribute less to the model performance is of significance to reducing monitoring costs.

## 5. Conclusions

In this work, XGBoost and LSTM models were used to forecast the concentration of cis-1,2-DCE and 1,1-DCE. XGBoost was more likely to capture DCE variation and be robust in high values, while the LSTM model presented better accuracies for all the wells. A well with a larger DCE concentration would cause a high error in the model’s predictive performance, and the influence of DCE concentration on XGBoost was more significant than that of LSTM. The explanation of the SHAP value of each variable indicated the high consistency with the rules of biodegradation in the real environment. ORP and conductivity were more important than temperature, pH, and DO in predicting. LSTM and XGBoost could realize the prediction of DCE concentrations with only water quality variables, and LSTM performed better than XGBoost in forecasting DCE.

## Figures and Tables

**Table 1:** The characteristics of selected wells (μg/L).

Well	Indicator	Tau	*p*-Value	Std	Mean	Range
JGW7-14m	cis-1,2-DCE	−0.81	3.21 × 10^−9^	56.11	75.08	12.7–225
JGW7-8m	cis-1,2-DCE	−0.87	3.23 × 10^−8^	52.45	78.16	11.6–175
JGW5-14m	cis-1,2-DCE	−0.65	1.13 × 10^−5^	210.84	204.75	13.4–826
JGW5-8m	cis-1,2-DCE	−0.54	3.63 × 10^−4^	215.58	194.27	13.2–664
JGW1-14m	cis-1,2-DCE	−0.65	3.49 × 10^−5^	849.37	1185.95	127–2840
JGW1-8m	cis-1,2-DCE	−0.64	1.62 × 10^−5^	1125.59	1273.33	127–3780
JC24-14m	1,1-DCE	−0.19	2.39 × 10^−1^	1021.69	662.29	5.6–3205
JC24-8m	1,1-DCE	−0.10	5.71 × 10^−1^	1128.40	516.47	3–4470
JC30-14m	1,1-DCE	−0.22	2.36 × 10^−1^	66.45	53.03	2–240
JC30-8m	1,1-DCE	−0.28	1.29 × 10^−1^	58.51	48.30	1.7–211
JC31-14m	1,1-DCE	−0.47	1.15 × 10^−2^	9.29	9.45	0.7–31.8
JC31-8m	1,1-DCE	−0.63	3.36 × 10^−4^	6.47	6.13	0.8–26.2
JC32-14m	1,1-DCE	−0.70	1.18 × 10^−5^	36,457.41	27,074.67	36.9–115,000
JC32-8m	1,1-DCE	−0.66	4.59 × 10^−5^	30,503.07	20,623.70	27.4–105,000
JC33-14m	1,1-DCE	−0.67	1.76 × 10^−4^	46.37	35.59	0.9–138.1
JC33-8m	1,1-DCE	−0.57	9.01 × 10^−4^	60.41	43.19	2–169
JC34-14m	1,1-DCE	−0.45	8.54 × 10^−3^	311.65	177.02	10.5–1390
JC34-8m	1,1-DCE	−0.48	5.14 × 10^−3^	285.68	170.98	10.8–1250

**Table 2:** Evaluation of predictive result of XGBoost and LSTM (mg/L).

Well	XGBoost			LSTM		
	RMSE	MAE	MAPE	RMSE	MAE	MAPE
cis-1,2-DCE						
JGW7-14m	0.08	0.076	4.30	0.052	0.050	2.80
JGW7-8m	0.081	0.078	4.80	0.071	0.069	4.20
JGW5-14m	0.29	0.25	8.60	0.11	0.096	3.80
JGW5-8m	0.25	0.25	9.50	0.18	0.14	5.00
JGW1-14m	1.10	1.10	5.30	0.76	0.67	3.60
JGW1-8m	2.30	2.30	9.30	0.53	0.44	2.20
Average	0.68	0.68	6.97	0.28	0.24	3.60
1,1-DCE						
JC24-14m	1.17	1.0010	24.40	1.35	0.77	3.91
JC24-8m	1.75	1.548	59.76	1.40	0.82	0.82
JC30-14m	0.077	0.052	4.26	0.080	0.055	4.03
JC30-8m	0.073	0.063	8.75	0.068	0.042	2.24
JC31-14m	0.0044	0.0036	2.49	0.0049	0.0045	2.72
JC31-8m	0.0043	0.0040	2.83	0.0037	0.0032	2.26
JC32-14m	57.46	54.68	563.40	26.54	21.07	253.60
JC32-8m	35.16	34.45	415.80	7.90	7.31	103.70
JC33-14m	0.041	0.038	17.62	0.013	0.011	5.37
JC33-8m	0.039	0.032	9.02	0.0074	0.0067	1.74
JC34-14m	0.18	0.16	6.46	0.094	0.076	2.97
JC34-8m	0.16	0.15	3.30	0.050	0.044	1.19
Average	8.01	7.68	93.18	3.13	2.52	32.05

## Data Availability

Not applicable.

## Supplementary Materials

The following supporting information can be downloaded at: https://www.mdpi.com/article/10.3390/ijerph19159374/s1, Supplementary Text S1: Long short-term memory (LSTM) model; Supplementary Text S2: Extreme gradient boosting (XGBoost) model; Supplementary Figure S1: Prediction results against measurement in JC32 (mg/L); Supplementary Figure S2: The SHAP values for each input variable in XGBoost; Supplementary Figure S3: The SHAP values for each input variable in LSTM; Supplementary Table S1: The quantitation limits of some organic compounds; Supplementary Table S2: Input variables of each well; Supplementary Table S3: Parameter in XGBoost; Supplementary Table S4: The detailed characteristics of variables in each selected well. References [20,23,24,36] are cited in the Supplementary Materials.
