# Supplementary material for: Prediction of Dichloroethene Concentration in the Groundwater of a Contaminated Site Using XGBoost and LSTM
Source: Int J Environ Res Public Health. 2022 Jul 30;19(15):9374. doi: 10.3390/ijerph19159374 (PMC9367752; doi:10.3390/ijerph19159374)
Supplement: Supplementary file 1 [file ijerph-19-09374-s001.zip › ijerph-1783679-supplementary.pdf]

Supplementary Material for

Prediction of dichloroethylene concentrations in the groundwater of contaminated sites Using LSTM and XGBoost

Feiyang Xia[1], Dengdeng Jiang[1], Lingya Kong[1], Yan Zhou[1], Da Ding[1], Yun Chen[1], Guoqing Wang[1], Shaopo Deng[1,*]

[1] State Environmental Protection Key Laboratory of Soil Environmental Management and Pollution Control, Nanjing Institute of Environmental Sciences, Ministry of Ecology and Environment of China, Nanjing, Jiangsu, China

* Corresponding authors.

E-mail: dengshaopo@foxmail.com (S. Deng)

TEL/FAX: +86- 25- 85287024 (S. Deng)

Text S1: Long short-term memory (LSTM) model

The Long Short-Term Memory neural networks were first proposed by Hochreiter and Schmidhuberin [1] to overcome the limitations (gradient vanishing and the exploding gradient problem) of recurrent neural networks (RNN) when predicting long-term sequential data. The main objective is to allow LSTM to learn long-term dependencies and save information for prolonged periods. The LSTM has a self-connection mechanism controlled by a multiplication gate that learns and decides when to clear the memory content by another unit [2]. The structure of the LSTM neural network is shown in Figure 2, which is comprised of different memory blocks called cells. The cell has three gates to learn and decide when to forget: the input gate, the output gate, and the forget gate. Forget gate is the first gate encountered by data, and it decides how much of the information should be discarded and it would forget the previous dependence and focus only on the newer dependence. The second gate is the input gate which decides what and how much information to remember. The output gate decides the output information in the current state. Two states are being transferred to the next cell, the cell state and the hidden state. With the function of three gates and two cell states, the LSTM model can update the cell unit at each time and learn the long period trend.

Forget gate: $f_t = \sigma(w_f s_t + U_f h_{t-1} + b_f)$

Input gate: $i_t = \sigma(w_i s_t + U_i h_{t-1} + b_i)$

Memory update: $\widetilde{C_t} = \tanh(w_c s_t + U_c h_{t-1} + b_c), C_t = f_t \otimes C_{t-1} + i_t \otimes \widetilde{C_t}$

Output gate: $O_t = \sigma(w_o s_t + U_o h_{t-1} + b_o)$

Hidden state: $h_t = O_t \otimes \tanh(C_t)$

where f_t, i_t *and* O_t are the sigmoid forget, input and output gates, respectively. Sigmoid function is a nonlinear activation function commonly used in machine learning. It can map a real value to the interval [0,1] to describe how much information passes

through [3]. w_x *and* U_c are parameters in LSTM. b_x are the bias vectors. $\otimes$ represents the multiplication of corresponding elements. C_t is the current cell state, and $\widetilde{C_t}$ is the new candidate values for the cell state. X_t are the current input data.

Text S2: Extreme Gradient Boosting (XGBoost) model [4].

XGBoost is an implementation of Gradient Boosted decision trees. In this algorithm, decision trees are composed of many weaker models which were in the sequential form. Weights are assigned to all the independent variables which are then fed into the decision tree which predicts results. The weight of variables predicted wrong by the tree is increased and these variables are then fed to the second decision tree.

The structure of one decision tree is:

$$\hat{y_i} = \sum_{k=1}^{k} f_k(X_i)$$

where, K is the number of trees, f is the predicted functional, i is the sample size. The objective function for the above model is given by:

$$obj(\theta) = \sum_{i}^{n} l(y_i, \hat{y_i}) + \sum_{k=1}^{k} \Omega(f_k)$$

where, $\sum_i^n l(y_i, \hat{y_i})$ is the loss function, y_i is the real value while $\hat{y_i}$ is the predicted value. $\sum_{k=1}^{k} \Omega(f_k)$ is the regularization parameter which showed the complexity of trees. The objective function of the above model can be defined as:

$$obj^{(t)} = \sum_{i=1}^{n} l(y_i, \hat{y_i}^{(t)}) + \sum_{i=1}^{t} \Omega(f_i)$$
$$= \sum_{i=1}^{n} l(y_i, \hat{y_i}^{(t-1)} + f_t(x_i)) + \Omega(f_t) + constant$$

Now, let's apply taylor series expansion to second order:

$$obj^{(t)} = \sum_{i=1}^{n}\left[l\left(y_i, y_i^{\wedge(t-1)}\right) + g_i f_t(x_i) + \frac{1}{2}h_i f_t^2(x_i)\right] + \Omega(f_t) + constant$$

Where $g_i = \partial_{y_i^{\wedge(t-1)}} l(y_i, y_i^{\wedge(t-1)})$, $h_i = \partial^2_{y_i^{\wedge(t-1)}} l(y_i, y_i^{\wedge(t-1)})$

$$f_t(x) = w_{q(x)}, w \in R^T, q: R^d \rightarrow \{1,2, \dots, T\}$$

Here, w is the vector of scores (weight) on leaves of tree, q is the function (structure of tree) assigning each data point to the corresponding leaf, and T is the number of leaves. The regularization term is then defined by:

$$\Omega(f_t) = \gamma T + \frac{1}{2}\lambda \sum_{j=1}^{T} w_j^2$$

Therefore, objective function becomes:

$$obj^{(t)} \approx \sum_{j=1}^{T}\left[g w_{q(x_i)} + \frac{1}{2}h_i w_{q(x_i)}^2\right] + \gamma T + \frac{1}{2}\lambda \sum_{j=1}^{T} w_j^2$$

$$= \left[(\sum_{i \in I_j} g_i) w_j + \frac{1}{2}(\sum_{i \in I_j} h_i + \lambda)\, w_j^2\right] + \gamma T$$

$$= \sum_{j=1}^{T}\left[G_j w_j + \frac{1}{2}(H_j + \lambda) w_j^2\right] + \gamma T$$

the best w_j for a given structure q(x) and the best objective reduction we can get is: $w_j' = -\frac{G_j}{H_j + \lambda}$, $obj = -\frac{1}{2}\sum_{j=1}^{T}\frac{G_j^2}{H_j + \lambda} + \gamma T$ where γ is pruning parameter, i.e. the least information gain to perform split. The smaller the objective function, the better the tree structure. But we can't directly optimize the tree, we will try to optimize one level of the tree at a time. Specifically, we try to split a leaf into two leaves, and the score it gains is:

$$Gain = \frac{1}{2}\left[\frac{G_L^2}{H_L + \lambda} + \frac{G_R^2}{R + \lambda} - \frac{(G_L + G_R)^2}{R + \lambda}\right] - \gamma$$

In order to find the best split of $Gain$, a extract greedy algorithm enumerates over all the possible splits on all the features.

Table S1. The quantitation limits of some organic compounds

Compounds	Methods	Quantitation limits (μg/L)
perchloroethene	USEPA 8260C-2006	0.5
Trichloroethene	USEPA 8260C-2006	0.5
1,1-dichloroethene	USEPA 8260C-2006	0.5
trans-1,2-dichloroethene	USEPA 8260C-2006	0.5
cis-1,2-dichloroethene	USEPA 8260C-2006	0.5
vinyl chloride	USEPA 8260C-2006	5
benzene	USEPA 8260C-2006	0.5
ethylbenzene	USEPA 8260C-2006	0.5
m,p-xylene	USEPA 8260C-2006	0.5
o-xylene	USEPA 8260C-2006	0.5
methylbenzene	USEPA 8260C-2006	0.5

Table S2. Input variables of each well

well	benzene	methylbenzene	ethylbenzene	m & p-xylene	o-xylene	vinyl chloride	pH	DO (mg/L)	temperature(°C)	ORP (mV)	conductivity (us/cm)	SUM
JGW1-8m	1	1	1	1	1	1	1	1	1	1	1	11
JGW1 14m	1	1	1	1	1	1	1	1	1	1	1	11
JGW5 8m	1	1	1	1	1	1	1	1	1	1	1	11
JGW5 14m	1	1	1	1	1	1	1	1	1	1	1	11
JGW7 8m	1	1	1	1	1	1	1	1	1	1	1	11
JGW7 14m	1	1	1	1	1	1	1	1	1	1	1	11
JC24 8m	1	1	1	1	1	1	1	1	1	1	1	11
JC24 14m	1	1	1	1	1	1	1	1	1	1	1	11
JC30 8m						1	1	1	1	1	1	6
JC30 14m							1	1	1	1	1	5
JC31 8m							1	1	1	1	1	5
JC31 14m							1	1	1	1	1	5
JC32 8m						1	1	1	1	1	1	6
JC32 14m						1	1	1	1	1	1	6
JC33 8m							1	1	1	1	1	5
JC33 14m							1	1	1	1	1	5
JC34 8m	1	1				1	1	1	1	1	1	8
JC34 14m	1	1				1	1	1	1	1	1	8

Table S3. Parameter in XGBoost

	max_depth	learning_rate	n_estimators
**JGW7-14m**	5	0.01	50
**JGW7-8m**	5	0.01	50
**JGW5-14m**	5	0.01	100
**JGW5-8m**	5	0.01	50
**JGW1-14m**	5	0.15	200
**JGW1-8m**	5	0.01	100
**JC24-14m**	10	0.01	100
**JC24-8m**	5	0.01	100
**JC30-14m**	5	2	50
**JC30-8m**	5	0.01	100
**JC31-14m**	5	0.05	50
**JC31-8m**	5	0.01	200
**JC32-14m**	5	0.01	100
**JC32-8m**	5	0.01	100
**JC33-14m**	5	0.01	100
**JC33-8m**	5	0.01	200
**JC34-14m**	5	0.01	200
**JC34-8m**	5	0.01	200

Table S4. The detailed characteristics of variables in each selected well

well		benzene	methylbenzene	ethylbenzene	m & p-xylene	o-xylene	vinyl chloride	pH	DO(mg/L)	temperature(°C)	ORP(mV)	conductivity(us/cm)	DCE
JGW1-8m	**count**	21.00	21.00	21.00	21.00	21.00	21.00	21.00	21.00	21.00	21.00	21.00	21.00
	**mean**	657.69	1512.23	10774.83	7768.86	839.29	2463.19	7.06	1.84	19.87	-30.98	7543.62	1273.33
	**std**	514.33	1043.09	8075.29	5769.21	479.24	2630.68	0.42	2.41	2.50	199.60	14646.70	1153.39
	**min**	106.00	11.60	7.40	331.00	107.00	129.00	6.14	0.05	16.43	-271.50	131.60	127.00
	**25%**	196.00	793.00	4160.00	3640.00	525.00	874.00	6.95	0.09	18.74	-161.90	2348.90	290.50
	**50%**	470.50	1110.00	8640.00	5725.00	736.00	1640.00	7.17	0.23	19.82	-80.30	3698.20	786.00
	**75%**	874.50	2430.00	15400.00	11700.00	1200.00	2685.00	7.22	4.20	20.57	43.90	4750.00	2270.00
	**max**	1580.00	3520.00	29800.00	20000.00	1770.00	9510.00	7.87	6.80	28.27	499.10	67947.00	3780.00
JGW1-14m	**count**	21.00	21.00	21.00	21.00	21.00	21.00	21.00	21.00	21.00	21.00	21.00	21.00
	**mean**	1314.12	1501.36	8340.20	6410.71	672.57	3500.97	6.92	1.47	19.00	-12.45	6056.77	1185.95
	**std**	835.60	809.48	5923.21	4442.39	409.60	3279.42	0.40	2.01	1.21	209.26	5267.26	870.35
	**min**	190.00	425.00	6.20	1940.00	168.00	178.00	6.15	0.05	16.34	-274.30	89.50	127.00
	**25%**	540.00	786.00	4590.00	2400.00	363.00	1684.00	6.53	0.09	18.70	-128.50	3079.90	412.00
	**50%**	1310.00	1340.00	7600.00	4915.00	651.50	2630.00	7.03	0.18	19.18	-61.90	5116.10	1090.00
	**75%**	1880.00	1995.00	10600.00	8680.00	815.00	3363.50	7.12	3.21	19.66	49.70	5980.80	1680.00
	**max**	3440.00	3050.00	22800.00	16700.00	2000.00	13200.00	7.72	5.37	21.00	522.25	24490.00	2840.00
JGW5-8m	**count**	21.00	21.00	21.00	21.00	21.00	21.00	21.00	21.00	21.00	21.00	21.00	21.00
	**mean**	4802.26	324.75	317.61	480.94	123.23	746.88	6.96	2.38	20.05	-0.35	7001.76	194.27
	**std**	1962.73	333.14	497.35	638.14	122.38	547.09	0.43	2.83	3.44	170.81	6762.61	220.91
	**min**	2010.00	20.20	4.60	32.00	15.00	74.00	6.37	0.07	15.14	-250.40	146.20	13.20
	**25%**	3830.00	148.00	87.70	111.00	25.75	437.00	6.68	0.13	18.19	-103.00	1366.65	41.50
	**50%**	4750.00	210.00	233.00	221.00	90.00	638.00	6.76	0.94	19.55	-44.70	3190.70	83.00
	**75%**	5497.50	379.00	297.50	591.50	172.00	1060.00	7.35	4.41	21.46	34.35	11899.00	324.93

well		benzene	methylbenzene	ethylbenzene	m & p-xylene	o-xylene	vinyl chloride	pH	DO(mg/L)	temperature(°C)	ORP(mV)	conductivity(us/cm)	DCE
	max	10600.00	1450.00	1980.00	2630.00	451.00	2050.00	7.93	8.50	31.27	466.55	19857.00	664.00
JGW5-14m	count	21.00	21.00	21.00	21.00	21.00	21.00	21.00	21.00	21.00	21.00	21.00	21.00
	mean	5839.76	531.26	387.80	671.84	190.15	974.07	6.74	1.08	19.50	-19.43	8630.72	204.75
	std	3101.55	502.98	483.14	807.80	234.67	456.64	0.41	1.78	1.70	159.28	5790.37	216.05
	min	1680.00	29.00	2.60	25.10	7.50	176.00	6.21	0.06	15.03	-268.30	296.00	13.40
	25%	4150.00	183.00	74.00	160.00	42.30	709.00	6.49	0.09	18.48	-97.60	4705.40	69.70
	50%	5660.00	452.00	249.00	462.00	79.00	901.00	6.65	0.17	20.04	-31.80	6839.30	110.00
	75%	6670.00	667.00	475.00	827.00	284.50	1110.00	6.91	1.06	20.75	-21.30	13935.10	227.00
	max	16900.00	2090.00	2020.00	3600.00	1010.00	2100.00	7.78	6.09	21.65	479.15	19175.80	826.00
JGW7-8m	count	21.00	21.00	21.00	21.00	21.00	21.00	21.00	21.00	21.00	21.00	21.00	21.00
	mean	2279.00	711.44	57.79	26.76	24.83	57.89	7.40	2.40	19.78	-53.05	4883.41	78.16
	std	2645.37	1663.47	44.22	34.29	8.02	26.33	0.84	2.73	3.35	198.80	8111.43	53.74
	min	285.00	1.20	14.20	2.40	12.30	26.00	6.14	0.02	14.10	-378.90	1652.15	11.60
	25%	660.00	8.10	31.00	6.66	20.15	40.33	6.97	0.10	17.54	-129.70	2333.63	31.00
	50%	1320.00	42.42	52.50	11.43	24.39	50.67	7.42	0.87	19.27	-77.30	2414.50	76.00
	75%	2990.00	206.00	68.30	35.30	28.83	66.33	7.74	3.58	22.76	55.00	2596.40	116.00
	max	11500.00	6770.00	202.00	140.00	47.50	132.21	10.19	7.59	26.93	351.40	34855.00	175.00
JGW7-14m	count	21.00	21.00	21.00	21.00	21.00	21.00	21.00	21.00	21.00	21.00	21.00	21.00
	mean	6695.48	873.91	67.11	54.48	37.41	221.48	7.15	1.79	19.06	-63.84	4581.90	75.08
	std	5062.39	1097.64	39.55	43.51	11.02	259.06	0.70	1.95	1.75	233.08	4164.20	57.49
	min	418.00	6.80	14.40	8.20	27.00	41.00	5.91	0.03	14.60	-375.70	1412.00	12.70
	25%	3016.00	173.00	39.26	23.10	31.11	90.00	6.76	0.09	18.08	-266.90	2546.33	28.05
	50%	5795.00	435.00	65.00	41.00	34.65	138.13	7.18	0.57	19.03	-90.70	2920.40	68.30
	75%	8640.00	933.50	89.30	62.40	37.40	235.25	7.47	2.90	20.47	73.00	3884.20	110.05
	max	18600.00	3585.85	153.00	174.00	67.90	1160.00	9.14	5.51	22.45	471.05	15066.50	225.00
JC34-8m	count	18.00	18.00	0.00	0.00	0.00	18.00	18.00	18.00	18.00	18.00	18.00	18.00
	mean	1.98	7.08				569.42	8.17	2.16	19.55	-4.02	2702.16	170.98

well		benzene	methylbenzene	ethylbenzene	m & p-xylene	o-xylene	vinyl chloride	pH	DO(mg/L)	temperature(°C)	ORP(mV)	conductivity(us/cm)	DCE
	**std**	1.54	14.94				810.45	0.70	2.84	2.53	151.50	294.47	293.97
	**min**	0.60	0.60				38.00	6.22	0.07	14.75	-234.00	1923.90	10.80
	**25%**	1.02	0.76				89.63	8.05	0.22	17.59	-121.00	2556.25	35.28
	**50%**	1.63	1.28				214.67	8.14	0.54	19.56	-20.00	2774.80	83.93
	**75%**	2.28	5.03				595.94	8.64	3.53	21.41	125.23	2930.55	120.50
	**max**	6.20	62.70				2730.00	9.20	8.70	23.73	225.10	3050.90	1250.00
JC34-14m	**count**	18.00	18.00	0.00	0.00	0.00	18.00	18.00	18.00	18.00	18.00	18.00	18.00
	**mean**	2.09	8.03				570.70	8.26	3.31	19.24	16.93	4267.78	177.02
	**std**	1.57	18.92				747.48	0.61	3.84	1.72	185.11	6456.50	320.68
	**min**	0.60	0.80				31.00	7.10	0.04	16.60	-257.00	1972.50	10.50
	**25%**	1.17	1.24				113.19	7.99	0.16	17.84	-69.70	2685.13	29.13
	**50%**	1.78	2.03				139.00	8.27	1.51	19.04	-9.75	2819.95	94.75
	**75%**	2.18	4.47				760.32	8.77	7.57	20.48	113.20	2961.43	131.35
	**max**	6.50	82.10				2110.00	9.17	10.17	23.22	370.10	30113.50	1390.00
JC33-8m	**count**	0.00	0.00	0.00	0.00	0.00	0.00	17.00	17.00	17.00	17.00	17.00	17.00
	**mean**							7.76	3.19	19.08	22.45	1237.93	43.19
	**std**							0.45	3.01	2.90	65.56	723.63	62.27
	**min**							6.56	0.04	14.76	-85.40	325.00	2.00
	**25%**							7.58	0.87	16.86	-18.00	678.98	3.00
	**50%**							7.80	1.89	19.23	7.00	958.70	6.20
	**75%**							8.03	6.20	20.56	59.00	1637.10	77.30
	**max**							8.38	8.90	23.70	199.90	2819.70	169.00
JC33-14m	**count**	0.00	0.00	0.00	0.00	0.00	0.00	17.00	17.00	17.00	17.00	17.00	17.00
	**mean**							7.69	2.83	18.94	35.59	1533.11	35.59
	**std**							0.56	2.46	2.21	59.68	1185.94	47.80
	**min**							6.33	0.00	14.94	-107.30	306.00	0.90
	**25%**							7.57	0.40	17.60	6.80	681.97	2.60

well		benzene	methylbenzene	ethylbenzene	m & p-xylene	o-xylene	vinyl chloride	pH	DO(mg/L)	temperature(°C)	ORP(mV)	conductivity(us/cm)	DCE
	50%							7.85	2.57	19.01	33.30	965.00	6.40
	75%							8.05	4.84	19.84	68.00	2582.85	62.20
	max							8.35	7.13	22.47	176.80	4200.70	138.10
JC32-8m	count	0.00	0.00	0.00	0.00	0.00	18.00	18.00	18.00	18.00	18.00	18.00	18.00
JC32-8m	mean						27527.83	7.94	2.96	19.72	63.55	2073.09	20623.70
	std						43539.80	0.38	3.22	2.22	62.60	1597.58	31387.40
	min						12.00	7.29	0.03	15.30	-17.00	379.00	27.40
	25%						441.00	7.75	0.52	17.93	29.90	975.30	375.38
	50%						4205.00	7.93	1.69	20.13	50.90	1496.75	5140.00
	75%						53325.00	8.15	5.10	20.92	87.18	3096.38	28025.00
	max						150000.00	8.54	9.70	22.81	207.20	5503.00	105000.00
JC32-14m	count	0.00	0.00	0.00	0.00	0.00	18.00	18.00	18.00	18.00	18.00	18.00	18.00
	mean						46475.56	7.83	3.14	19.14	79.19	2776.84	27074.67
	std						69181.99	0.36	3.09	1.03	89.24	2263.08	37514.36
	min						14.00	7.03	0.00	16.70	-40.40	395.00	36.90
	25%						434.75	7.62	0.76	18.53	27.00	822.18	267.75
	50%						3245.00	7.84	2.23	19.35	62.63	1740.95	6160.00
	75%						82278.13	7.95	4.88	19.61	93.66	4585.25	49350.00
	max						198000.00	8.44	9.40	21.09	263.20	7692.50	115000.00
JC31-8m	count	0.00	0.00	0.00	0.00	0.00	0.00	16.00	16.00	16.00	16.00	16.00	16.00
	mean							7.72	3.33	18.94	56.06	1635.85	6.13
	std							0.27	3.86	2.64	72.73	220.08	6.68
	min							7.28	0.12	14.62	-99.70	1240.50	0.80
	25%							7.57	0.39	16.99	17.65	1472.75	2.30
	50%							7.73	0.95	18.47	50.30	1672.13	4.50
	75%							7.86	7.51	20.54	105.65	1794.90	6.63
	max							8.14	9.40	23.80	174.80	2032.20	26.20

well		benzene	methylbenzene	ethylbenzene	m & p-xylene	o-xylene	vinyl chloride	pH	DO(mg/L)	temperature(°C)	ORP(mV)	conductivity(us/cm)	DCE
JC31-14m	**count**	0.00	0.00	0.00	0.00	0.00	0.00	16.00	16.00	16.00	16.00	16.00	16.00
	**mean**							7.62	3.43	19.16	58.42	1630.74	9.45
	**std**							0.23	4.39	1.74	69.05	218.94	9.60
	**min**							7.18	0.05	16.26	-82.20	1252.50	0.70
	**25%**							7.54	0.27	17.79	12.26	1474.98	3.38
	**50%**							7.60	0.75	18.77	55.25	1651.50	5.90
	**75%**							7.69	6.91	20.24	120.70	1782.70	13.81
	**max**							8.10	11.68	22.60	164.60	2023.90	31.80
JC30-8m	**count**	0.00	0.00	0.00	0.00	0.00	0.00	17.00	17.00	17.00	17.00	17.00	17.00
	**mean**							8.22	3.81	19.74	54.11	1767.17	48.30
	**std**							0.60	3.24	2.35	88.58	2321.08	60.31
	**min**							6.68	0.18	16.21	-65.40	1.52	1.70
	**25%**							8.08	0.50	18.08	8.10	1077.30	11.20
	**50%**							8.17	3.88	19.55	36.00	1294.30	21.60
	**75%**							8.44	6.45	21.38	83.45	1367.10	46.90
	**max**							9.41	8.88	23.47	320.60	10622.00	211.00
JC30-14m	**count**	0.00	0.00	0.00	0.00	0.00	17.00	17.00	17.00	17.00	17.00	17.00	17.00
	**mean**						43.35	8.28	3.16	18.79	60.24	1318.23	53.03
	**std**						20.91	0.50	2.89	1.73	57.71	458.04	68.49
	**min**						11.00	7.33	0.07	16.50	3.00	734.21	2.00
	**25%**						27.50	7.96	0.30	17.65	22.00	1061.40	16.90
	**50%**						45.00	8.31	3.69	18.52	36.60	1295.00	26.70
	**75%**						52.60	8.42	6.30	20.00	87.40	1404.60	33.20
	**max**						92.00	9.43	7.70	23.27	225.20	2685.20	240.00
JC24-8m	**count**	21.00	21.00	21.00	21.00	21.00	21.00	21.00	21.00	21.00	21.00	21.00	21.00
	**mean**	13.45	10.62	8.79	8.47	18.01	1887.94	8.27	2.30	19.25	0.02	5714.82	516.47
	**std**	10.14	13.83	8.84	8.10	8.14	4416.09	0.60	2.64	1.99	148.72	14460.49	1156.27

well		benzene	methylbenzene	ethylbenzene	m & p-xylene	o-xylene	vinyl chloride	pH	DO(mg/L)	temperature(°C)	ORP(mV)	conductivity(us/cm)	DCE
	**min**	5.60	0.70	1.10	1.80	1.90	7.00	7.42	0.09	14.87	-207.60	14.10	3.00
	**25%**	7.97	3.20	5.85	3.05	15.40	70.00	7.89	0.29	18.12	-99.60	1477.30	11.60
	**50%**	11.10	6.30	7.40	6.79	17.73	247.01	8.16	0.92	19.00	-7.70	1658.00	52.40
	**75%**	13.90	13.00	8.90	9.60	24.10	766.00	8.74	3.36	20.22	35.75	1993.10	270.05
	**max**	53.80	64.00	45.30	31.80	35.66	15100.00	9.71	8.50	23.03	357.85	63608.00	4470.00
JC24-14m	**count**	21.00	21.00	21.00	21.00	21.00	21.00	21.00	21.00	21.00	21.00	21.00	21.00
	**mean**	24.13	13.63	10.71	9.48	17.24	18829.47	8.26	2.11	19.01	-20.90	3947.88	662.29
	**std**	37.74	26.77	15.71	12.50	9.39	43027.47	0.49	2.78	1.48	147.63	10373.63	1046.92
	**min**	2.70	0.90	1.05	1.73	2.69	12.00	7.46	0.10	15.93	-215.70	32.20	5.60
	**25%**	9.20	2.50	5.31	2.80	12.05	823.00	7.85	0.23	18.40	-101.70	1507.20	22.60
	**50%**	11.20	5.60	8.10	6.53	15.50	2320.00	8.24	0.76	19.02	-36.00	1674.75	125.00
	**75%**	21.00	12.13	10.10	9.88	20.24	7590.00	8.57	2.74	19.38	25.10	1842.10	713.00
	**max**	180.00	126.00	77.80	58.60	38.90	172000.00	9.07	9.00	22.21	381.75	49150.00	3205.00

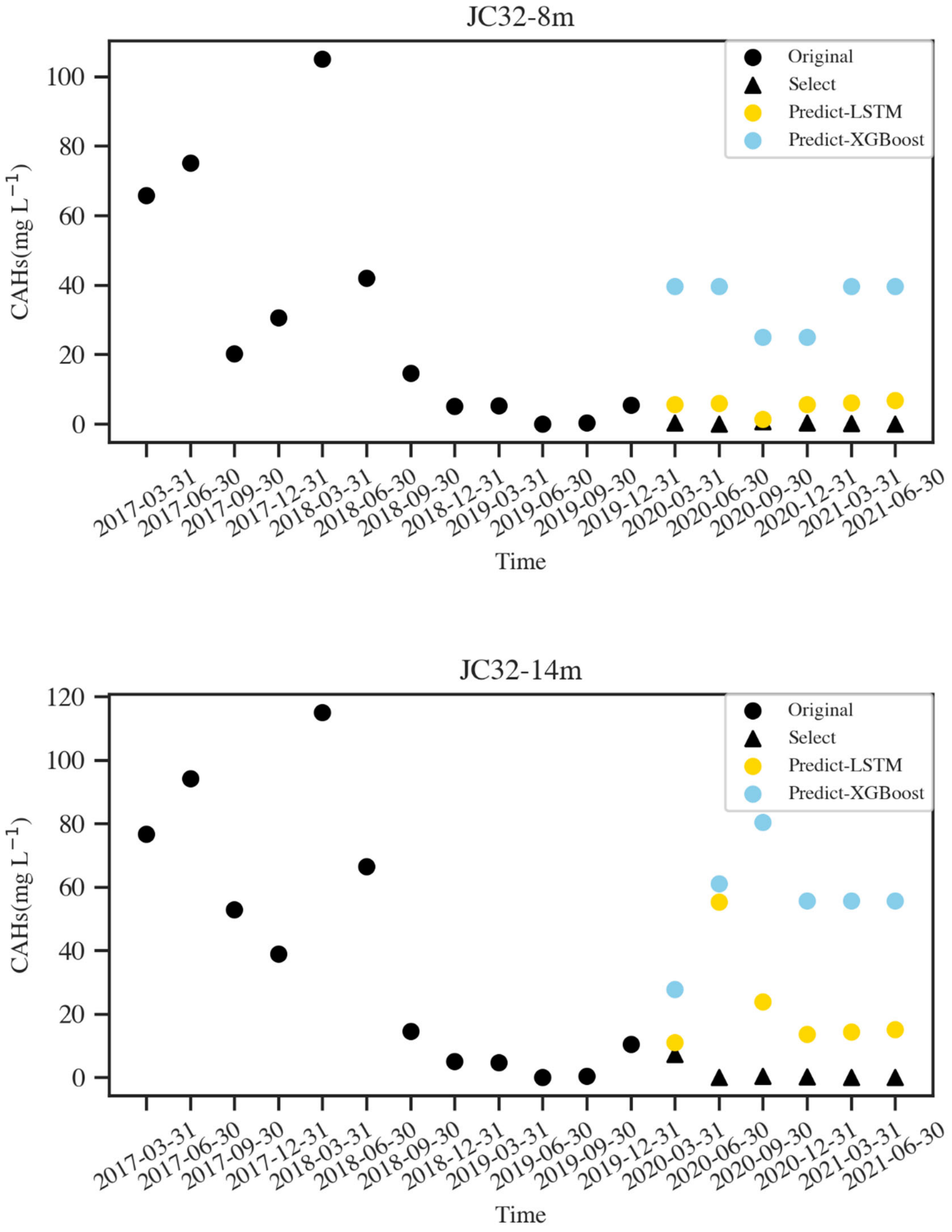

Figure S1. Prediction results against measurement in JC32 (mg/L)

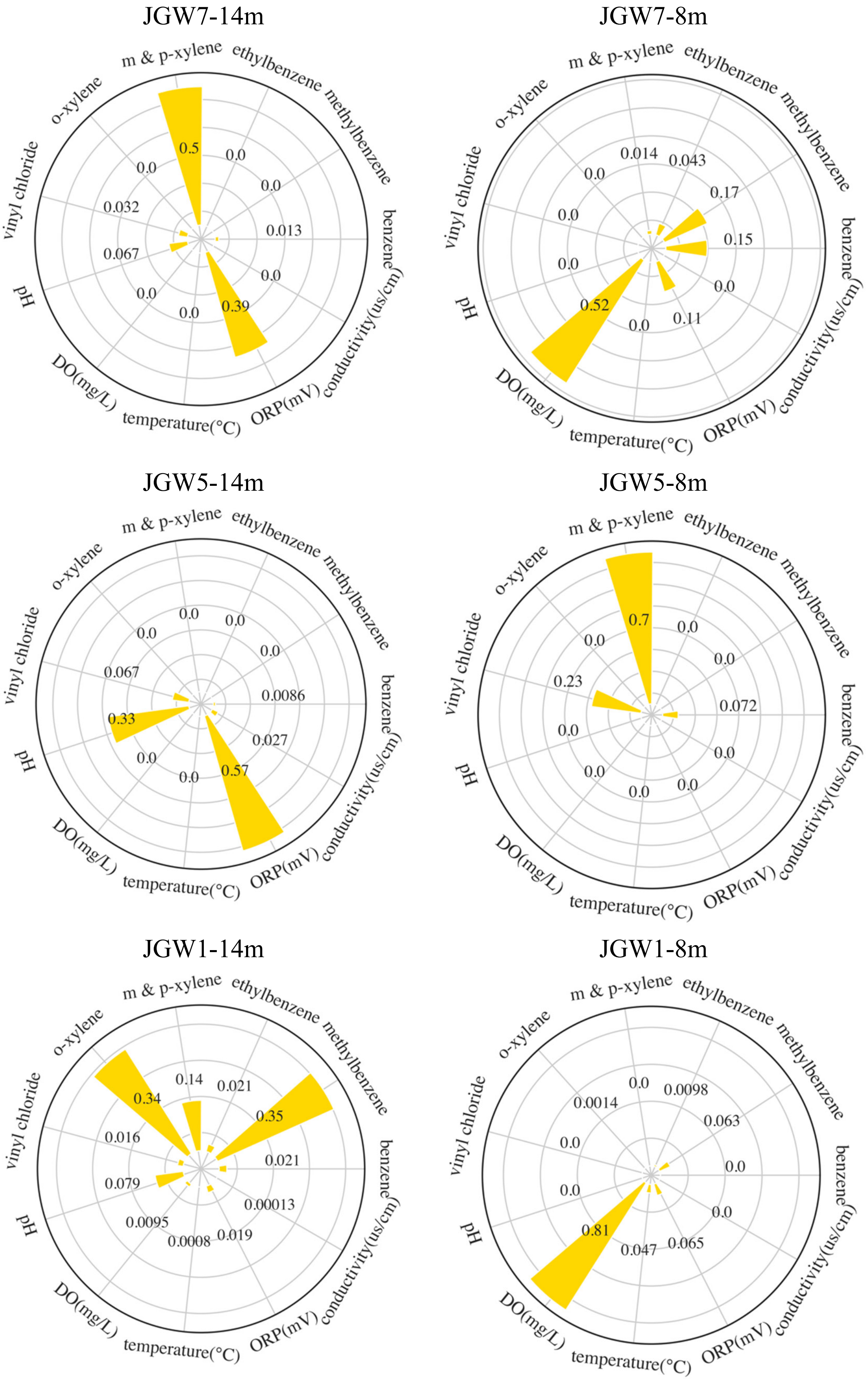

JGW7-14m
m & p-xylene
ethylbenzene
methylbenzene
benzene
conductivity(us/cm)
ORP(mV)
temperature(°C)
DO(mg/L)
pH
vinyl chloride
o-xylene
0.5
0.0
0.0
0.013
0.0
0.39
0.0
0.0
0.067
0.032
0.0
JGW7-8m
m & p-xylene
ethylbenzene
methylbenzene
benzene
conductivity(us/cm)
ORP(mV)
temperature(°C)
DO(mg/L)
pH
vinyl chloride
o-xylene
0.014
0.043
0.17
0.15
0.0
0.11
0.0
0.52
0.0
0.0
0.0
JGW5-14m
m & p-xylene
ethylbenzene
methylbenzene
benzene
conductivity(us/cm)
ORP(mV)
temperature(°C)
DO(mg/L)
pH
vinyl chloride
o-xylene
0.0
0.0
0.0
0.0086
0.027
0.57
0.0
0.0
0.33
0.067
0.0
JGW5-8m
m & p-xylene
ethylbenzene
methylbenzene
benzene
conductivity(us/cm)
ORP(mV)
temperature(°C)
DO(mg/L)
pH
vinyl chloride
o-xylene
0.7
0.0
0.0
0.072
0.0
0.0
0.0
0.0
0.0
0.23
0.0
JGW1-14m
m & p-xylene
ethylbenzene
methylbenzene
benzene
conductivity(us/cm)
ORP(mV)
temperature(°C)
DO(mg/L)
pH
vinyl chloride
o-xylene
0.14
0.021
0.35
0.021
0.00013
0.019
0.0008
0.0095
0.079
0.016
0.34
JGW1-8m
m & p-xylene
ethylbenzene
methylbenzene
benzene
conductivity(us/cm)
ORP(mV)
temperature(°C)
DO(mg/L)
pH
vinyl chloride
o-xylene
0.0
0.0098
0.063
0.0
0.0
0.065
0.047
0.81
0.0
0.0
0.0014

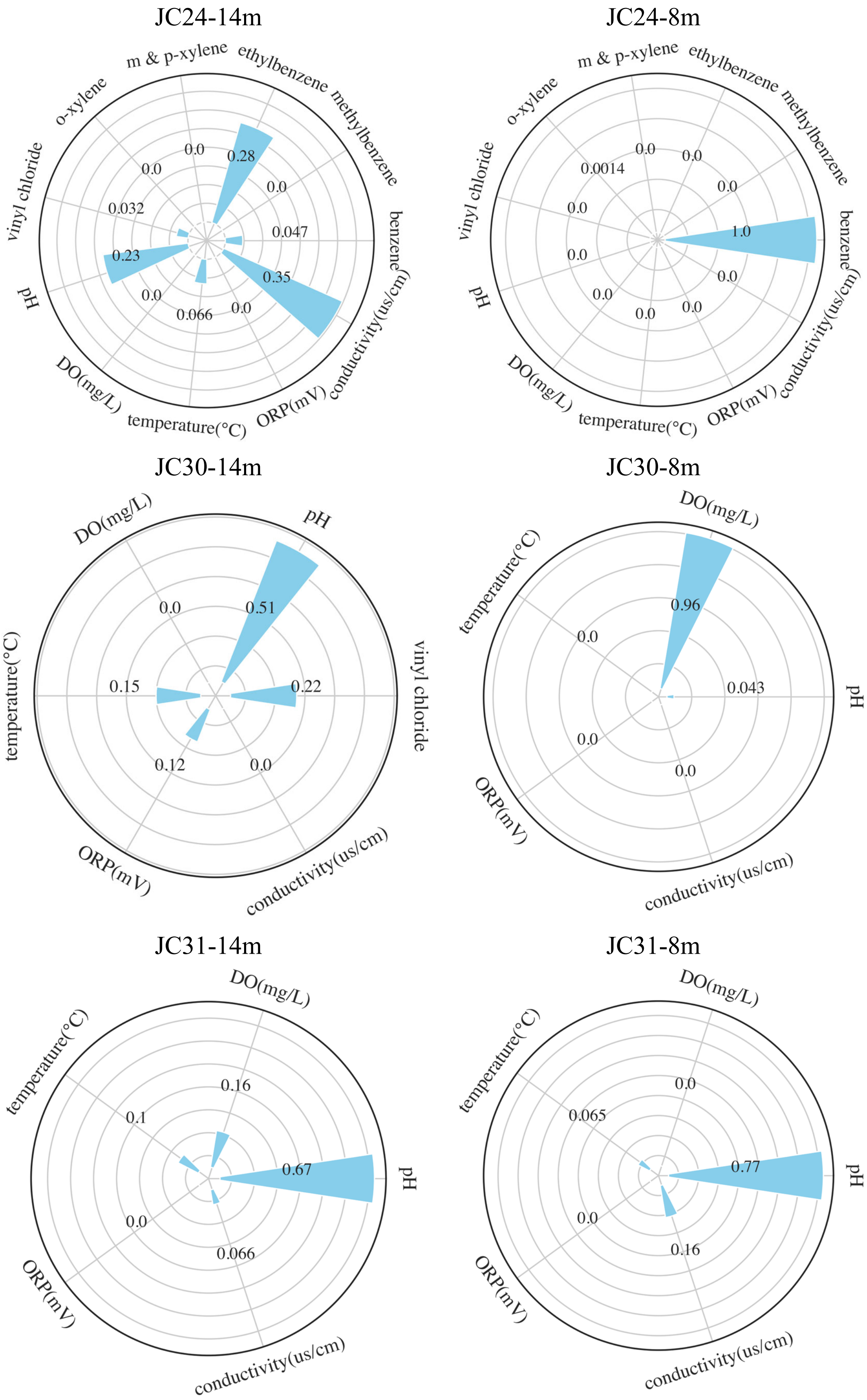
JC24-14m
m & p-xylene
ethylbenzene
methylbenzene
benzene
conductivity(us/cm)
ORP(mV)
temperature(°C)
DO(mg/L)
pH
vinyl chloride
o-xylene
0.0
0.28
0.0
0.0
0.047
0.032
0.23
0.35
0.0
0.066
0.0
JC24-8m
m & p-xylene
ethylbenzene
methylbenzene
benzene
conductivity(us/cm)
ORP(mV)
temperature(°C)
DO(mg/L)
pH
vinyl chloride
o-xylene
0.0
0.0
0.0014
0.0
0.0
1.0
0.0
0.0
0.0
0.0
0.0
JC30-14m
DO(mg/L)
pH
vinyl chloride
conductivity(us/cm)
ORP(mV)
temperature(°C)
0.0
0.51
0.15
0.22
0.12
0.0
JC30-8m
DO(mg/L)
pH
conductivity(us/cm)
ORP(mV)
temperature(°C)
0.96
0.0
0.043
0.0
0.0
JC31-14m
DO(mg/L)
pH
conductivity(us/cm)
ORP(mV)
temperature(°C)
0.16
0.1
0.67
0.0
0.066
JC31-8m
DO(mg/L)
pH
conductivity(us/cm)
ORP(mV)
temperature(°C)
0.0
0.065
0.77
0.0
0.16

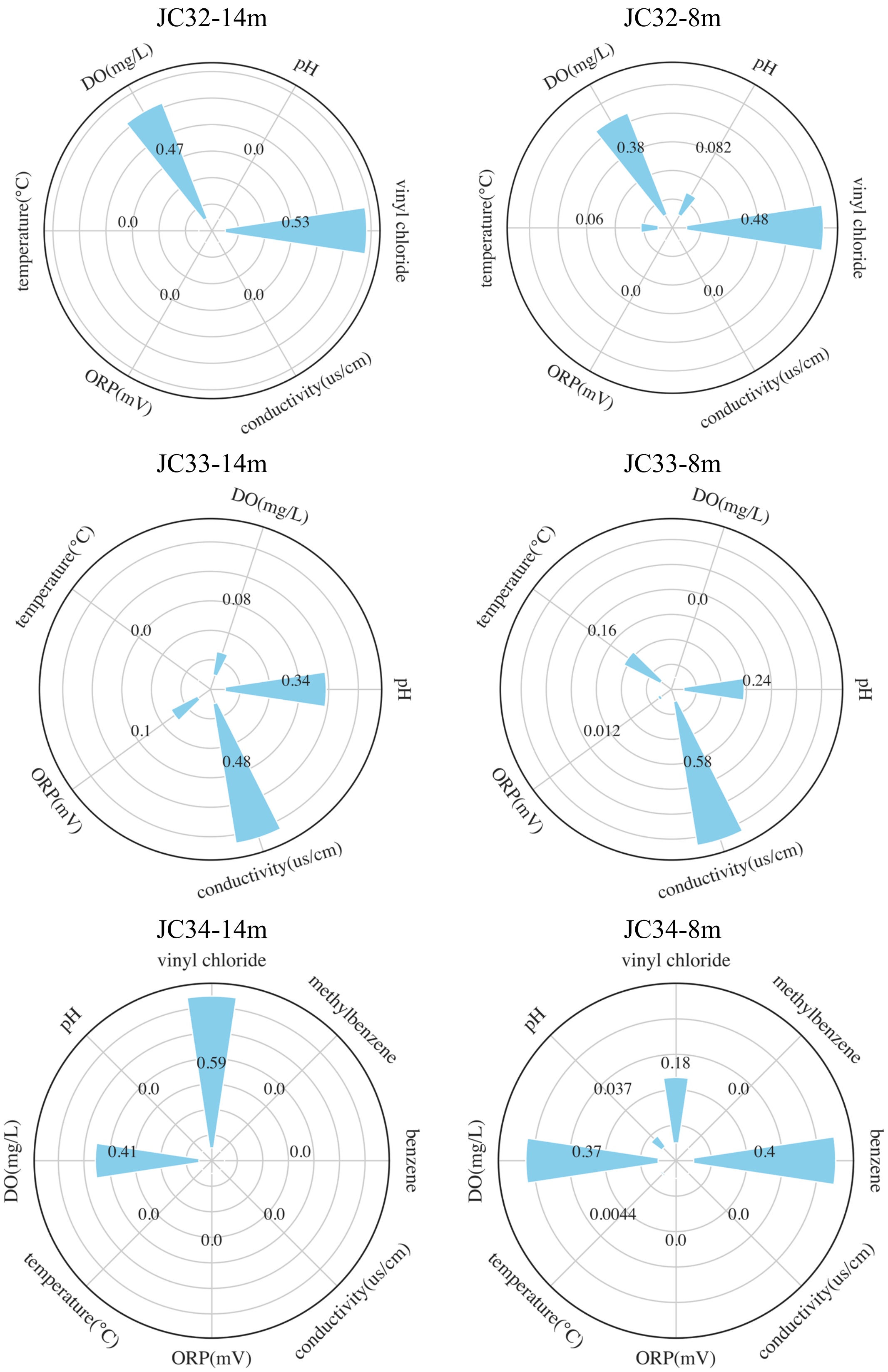

Figure S2. The SHAP values for each input variable in XGBoost

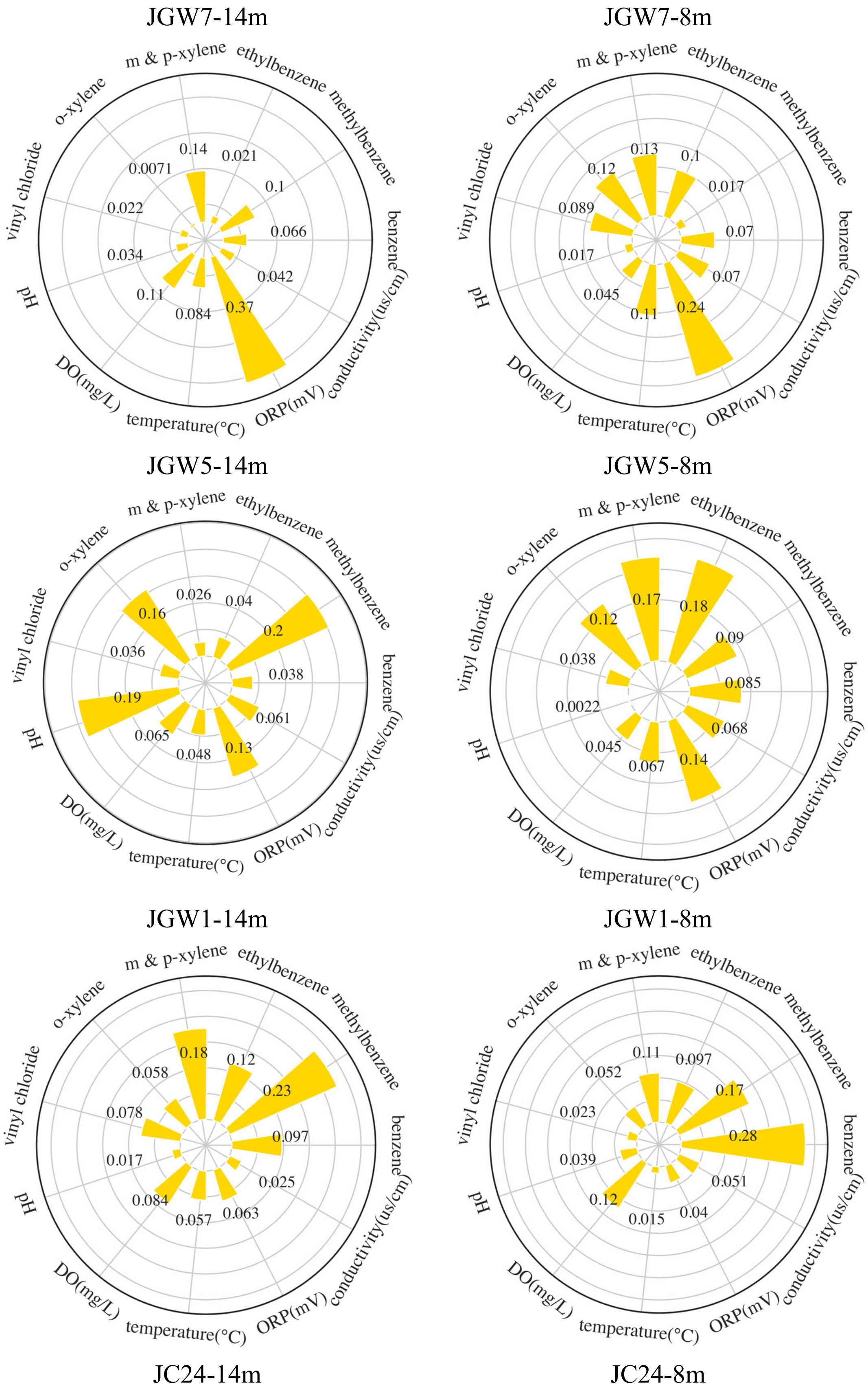

JGW7-14m
m & p-xylene
ethylbenzene
methylbenzene
benzene
conductivity(us/cm)
ORP(mV)
temperature(°C)
DO(mg/L)
pH
vinyl chloride
o-xylene
0.14
0.021
0.0071
0.1
0.022
0.066
0.034
0.042
0.11
0.084
0.37
JGW7-8m
0.13
0.1
0.12
0.017
0.089
0.07
0.017
0.07
0.045
0.11
0.24
JGW5-14m
0.026
0.04
0.16
0.2
0.036
0.038
0.19
0.061
0.065
0.048
0.13
JGW5-8m
0.17
0.18
0.12
0.09
0.038
0.085
0.0022
0.068
0.045
0.067
0.14
JGW1-14m
JGW1-8m
0.18
0.12
0.058
0.23
0.078
0.097
0.017
0.025
0.084
0.057
0.063
0.11
0.097
0.052
0.17
0.023
0.28
0.039
0.051
0.12
0.015
0.04
JC24-14m
JC24-8m

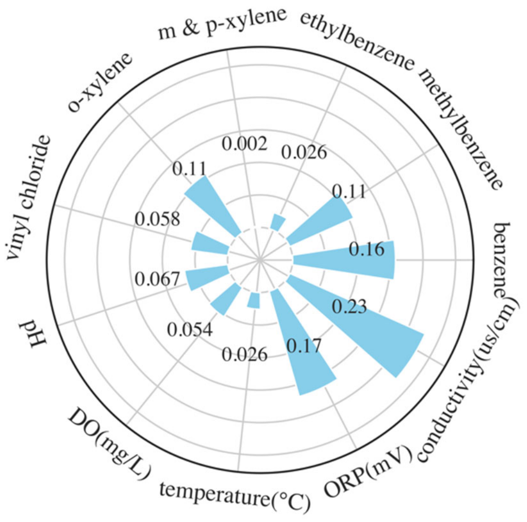

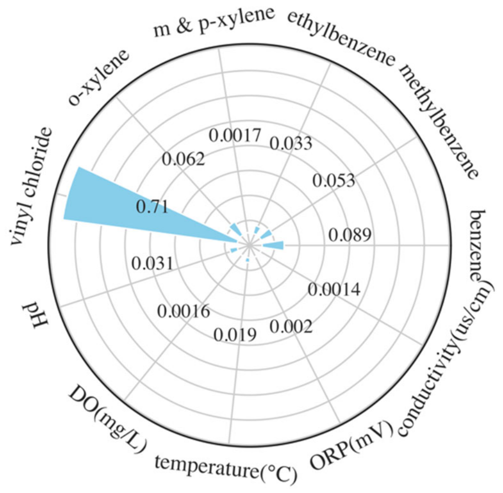

JC30-14m

JC30-8m

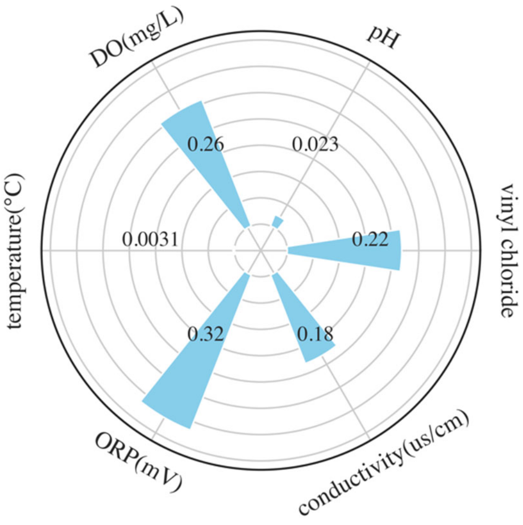

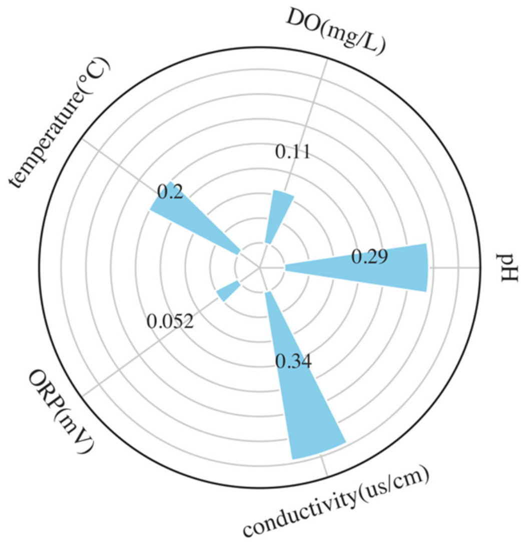

JC31-14m

JC31-8m

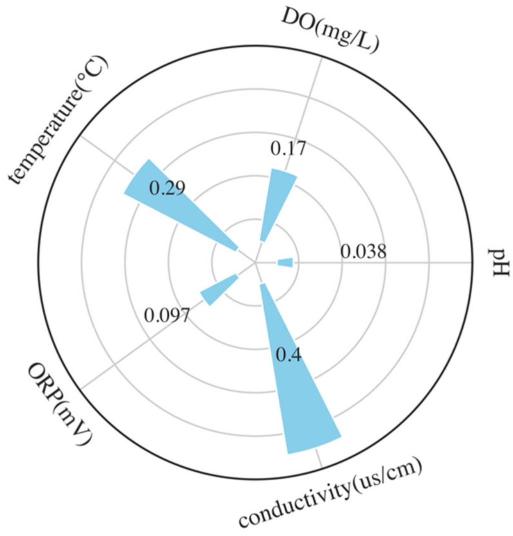

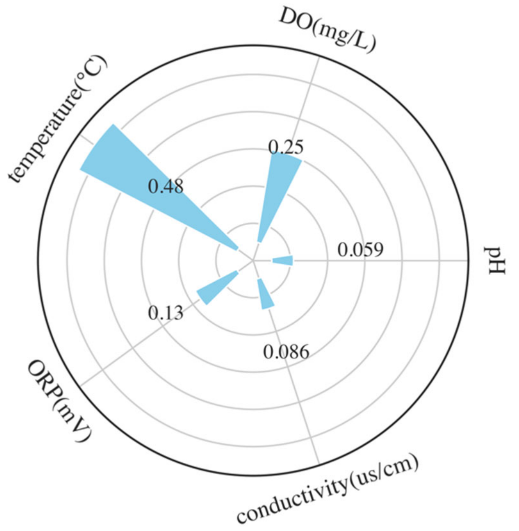

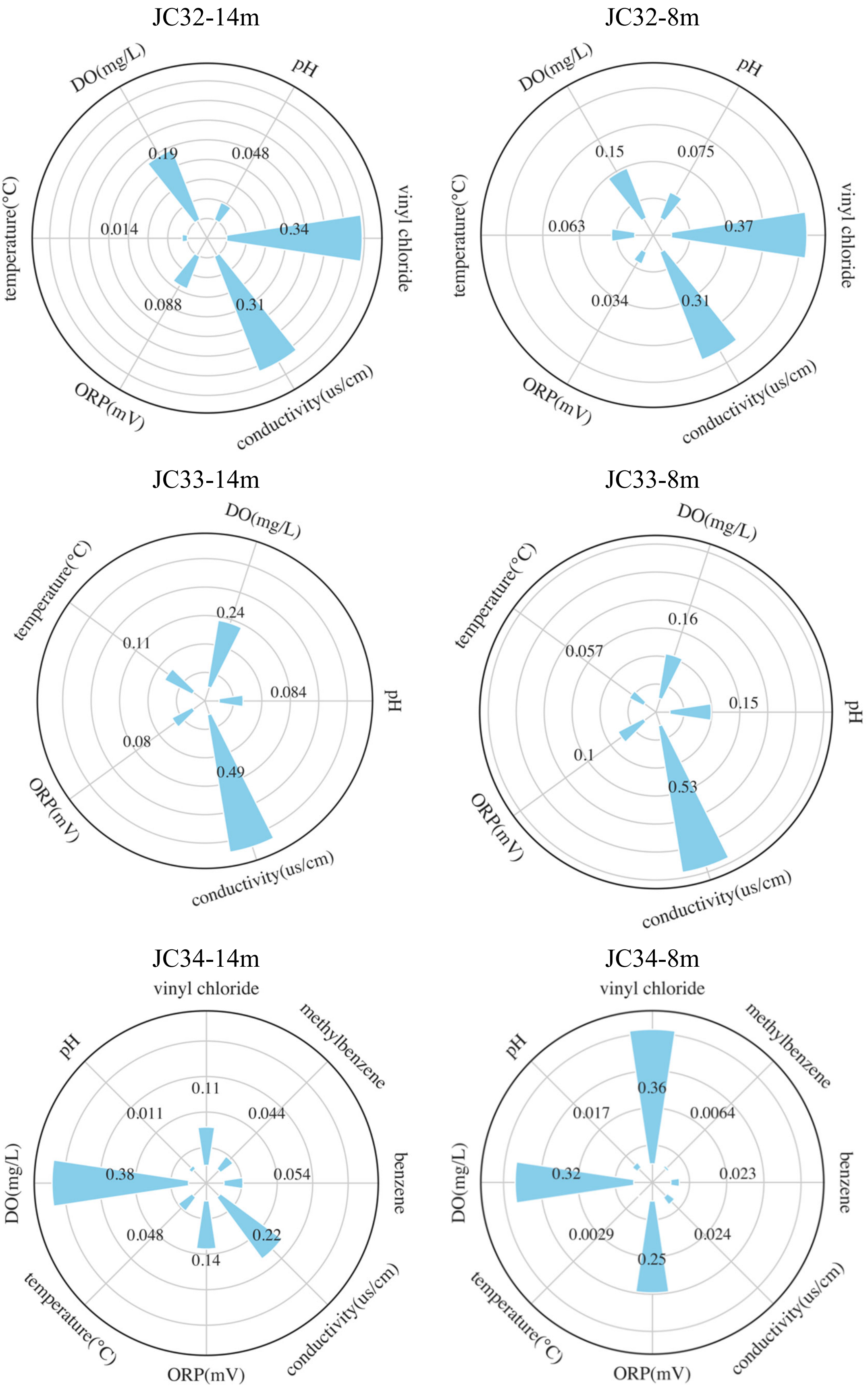

Figure S3. The SHAP values for each input variable in LSTM

Reference

[1] Cubillos, M. Multi-site household waste generation forecasting using a deep learning approach. Waste Manag. 2020, 115, 8-14. Doi:10.1016/j.wasman.2020.06.046
[2]Chen, T., & Guestrin, C. *XGBoost*. Paper presented at the Proceedings of the 22nd ACM SIGKDD International Conference on Knowledge Discovery and Data Mining. 2016
[3] Hochreiter, S.; Schmidhuber, J. Long short-term memory. Neural Comput. 1997, 9 (8), 1735–1780. Doi:10.1162/neco.1997.9.8.1735.
[4] Man Y.; Yang Q.; Shao J.; Wang G.; Bai L.; Xue Y. Enhanced LSTM model for daily runoff prediction in the upper huai river basin, china. Engineering, 2022 (in press). Doi:10.1016/j.eng.2021.12.022
